# Cult, herding, and ‘pilgrimage’ in the Late Neolithic of north-west Arabia: Excavations at a mustatil east of AlUla

**DOI:** 10.1371/journal.pone.0281904

**Published:** 2023-03-15

**Authors:** Melissa Kennedy, Laura Strolin, Jane McMahon, Daniel Franklin, Ambika Flavel, Jacqueline Noble, Lauren Swift, Ahmed Nassr, Stewart Fallon, Hugh Thomas

**Affiliations:** 1 Classics and Ancient History, University of Western Australia, Perth, Australia; 2 Museum of Natural History of Geneva, Geneva, Switzerland; 3 Forensic Anthropology, University of Western Australia, Perth, Australia; 4 Department of Tourism and Archaeology, College of Arts, University of Ha’il, Ha’il, Saudi Arabia; 5 Research School of Earth Sciences, College of Science, Australian National University, Canberra, Australia; Griffith University, AUSTRALIA

## Abstract

Since the 1970s, monumental stone structures now called mustatil have been documented across Saudi Arabia. However, it was not until 2017 that the first intensive and systematic study of this structure type was undertaken, although this study could not determine the precise function of these features. Recent excavations in AlUla have now determined that these structures fulfilled a ritual purpose, with specifically selected elements of both wild and domestic taxa deposited around a betyl. This paper outlines the results of the University of Western Australia’s work at site IDIHA-0008222, a 140 m long mustatil (IDIHA-F-0011081), located 55 km east of AlUla. Work at this site sheds new and important light on the cult, herding and ‘pilgrimage’ in the Late Neolithic of north-west Arabia, with the site revealing one of the earliest chronometrically dated betyls in the Arabian Peninsula and some of the earliest evidence for domestic cattle in northern Arabia.

## Introduction

Since the 1970s, monumental structures now called mustatil (previously known as ‘gates’) have been documented across Saudi Arabia [1–3]. Mustatil is the Arabic for rectangle (مستطيل), plural has been anglicised to mustatils. However, it was not until 2017 that the first intensive and systematic study of this structure type was undertaken by David Kennedy [4]. Kennedy’s [4] study was based on remote sensing data and was focused primarily upon the Harrat Khaybar and areas to the east. Due to the nature of the data-set, Kennedy was unable to hypothesise a precise function for these enigmatic structures. However, subsequent studies based on ground survey and preliminary excavation data revealed that the mustatil served a ritual purpose during the Arabian Late Neolithic [5–7]. In 2018, under the auspices of the Royal Commission for AlUla, the first mustatil was excavated by Wael Abu-Azizeh with Oxford Archaeology, with these excavations revealing offering chambers with *in situ* ritual faunal deposits [7]. Over the course of the 2019 and 2020 excavation seasons the University of Western Australia’s AAKSA project began excavating another mustatil 55 km east of the modern city of AlUla. Excavations at this site (IDIHA-0008222) revealed a series of ritual and mortuary deposits, that shed new light on the adoptions of domestic cattle, cult and ‘pilgrimage’ in the Late Neolithic of north-west Arabia.

### The Mustatils of Northern Arabia

**Fig 1:**
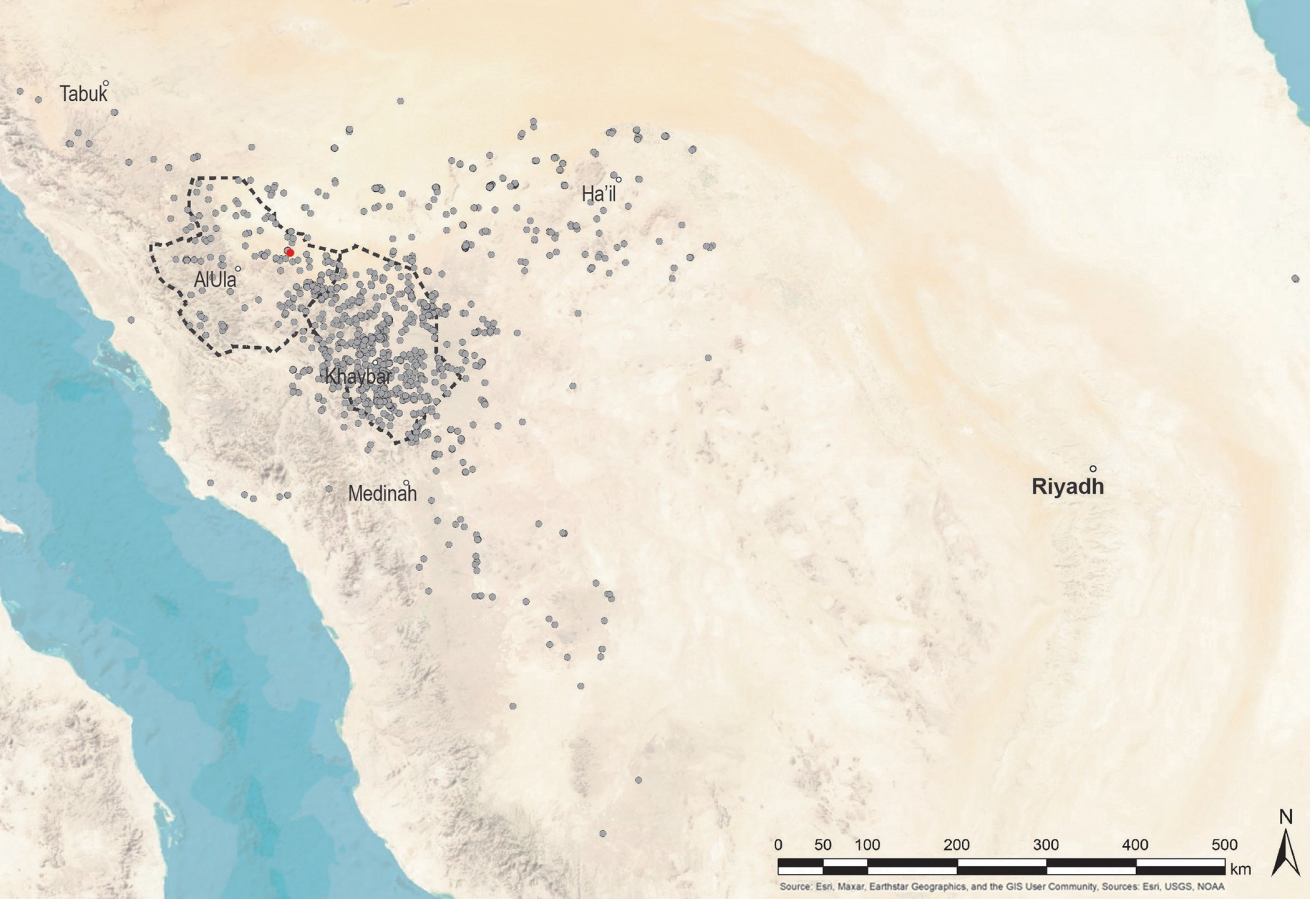
Mustatil locations in Saudi Arabia, location of site IDIHA-0008222 marked in red. Source: Esri, Maxar, Earthstar Geographics, and the GIS User Community, Sources: Esri, USGS, NOAA.

**Fig 2:**
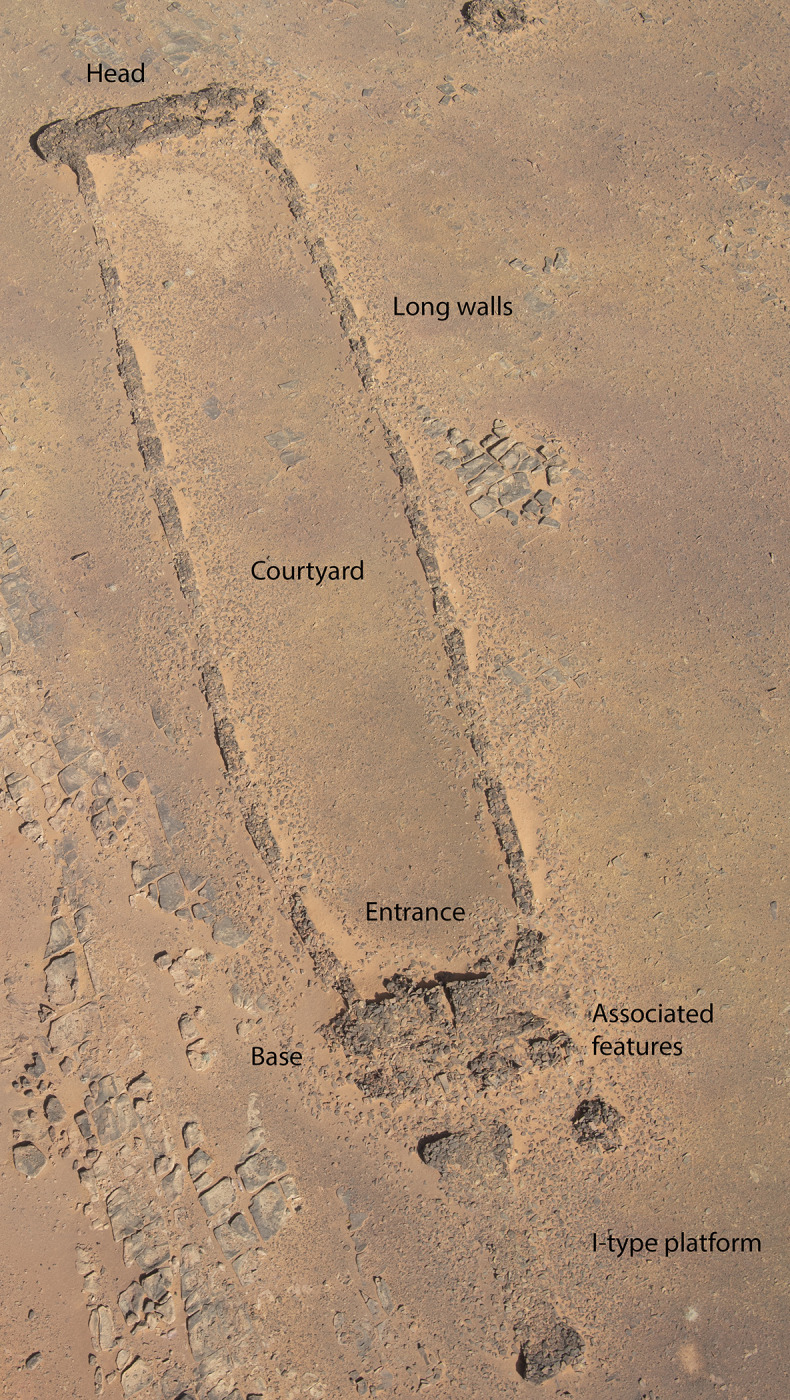
Main architectural features of a mustatil (IDIHA-F-0003301.

Concentrated in northern Arabia, over 1600 mustatils have been identified through remote sensing, aerial photography, and ground survey [6] (Fig 1). Ranging from 20-600m in length, these monumental structures were constructed from locally available stone, such as sandstone desert pavement or basalt. Distinguished by a rectangular to sub-rectangular form; mustatils are characterised by two parallel platforms (head and base; Fig 2); linked by perpendicularly set long-walls, creating a large open courtyard. Internal dividing walls, ranging in number from one to three have also been identified in the courtyards of some examples [6]. In addition to dividing walls, in front of the base, numerous instances included discrete or interlocking cells, often associated with orthostats. Survey work has also identified narrow entranceways in the base platform, and chambers in the head [6]. The excavated contents of these chambers have suggested that they were the focus of ritual activity, with current survey evidence indicating that the chambers were both sealed and open-air [6]. To date, limited radiocarbon evidence has been presented for these structures, with available assays indicating a Middle Holocene, Late Neolithic date (6^th^ and 5^th^ millennia BCE) [5–7].

### IDIHA-0008222: Location and description

**Fig 3:**
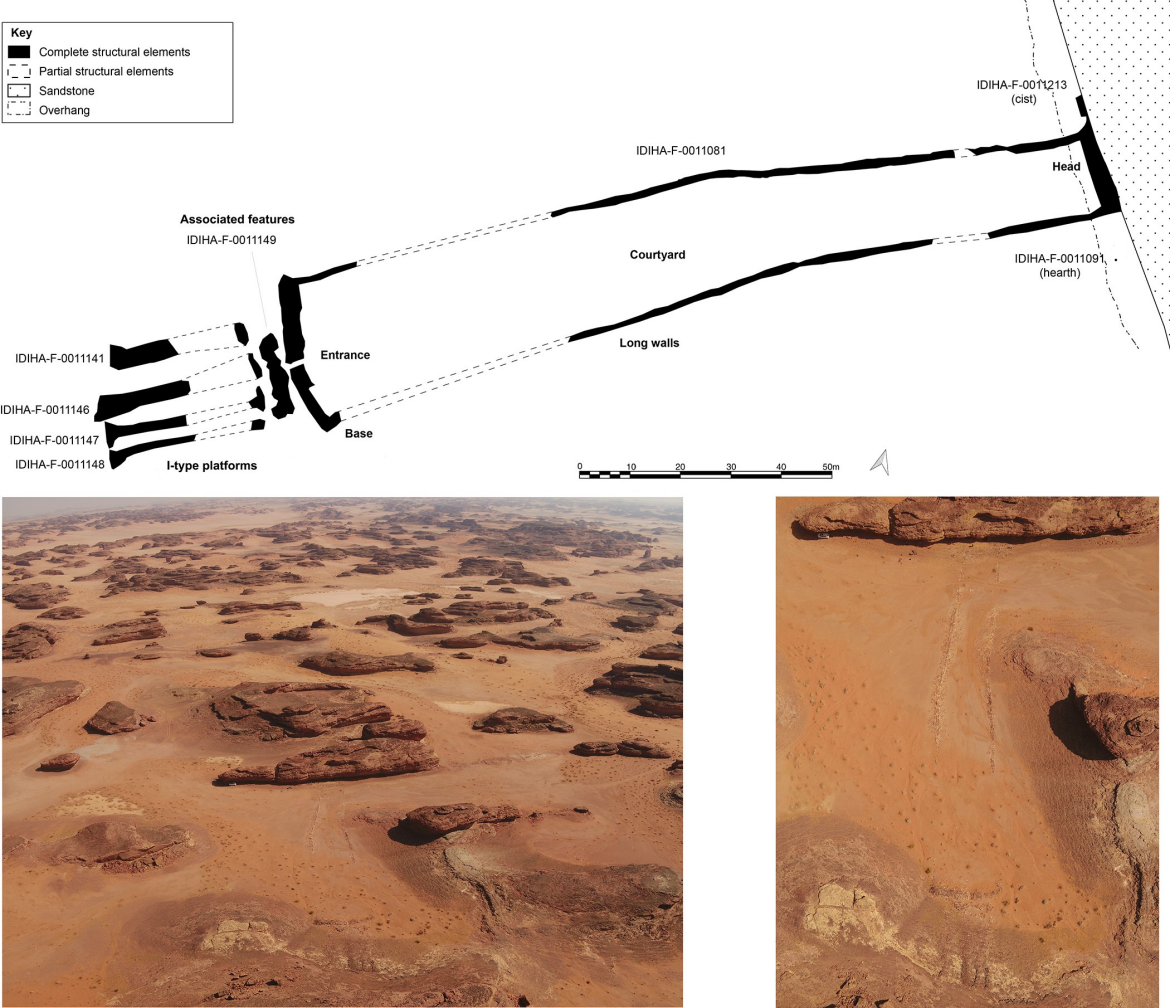
Top: Plan of site IDIHA-0008222. Bottom: Aerial photographs of IDIHA-0008222, not to scale, photos orientated east.

The site IDIHA-0008222 is located in a narrow valley in an area of dense sandstone canyons, 55 km east of the town of AlUla. The site consists of a 140m long complex mustatil (IDIHA-F-0011081), orientated roughly southwest-northeast (Fig 3). Constructed from locally available slabs of sandstone desert pavement, the mustatil is positioned between two north-south running yardangs. This low-walled structure has minimal visibility in the landscape, with a series of yardangs obscuring wider views to and from the structure until one is in the valley itself. The head of the structure is located in the north-east, and is one of the few examples in AlUla where the head is situated beneath a rock overhang, abutting the base of a yardang. The base is located in the south-west, with a 1m wide entranceway located in the centre. In the inter-yardang corridor, towards the head of the mustatil, the long walls are marked by a sudden shift in orientation, with this change potentially due to a projecting yardang. No identifiable architectural features were identified in the courtyard.

**Fig 4:**
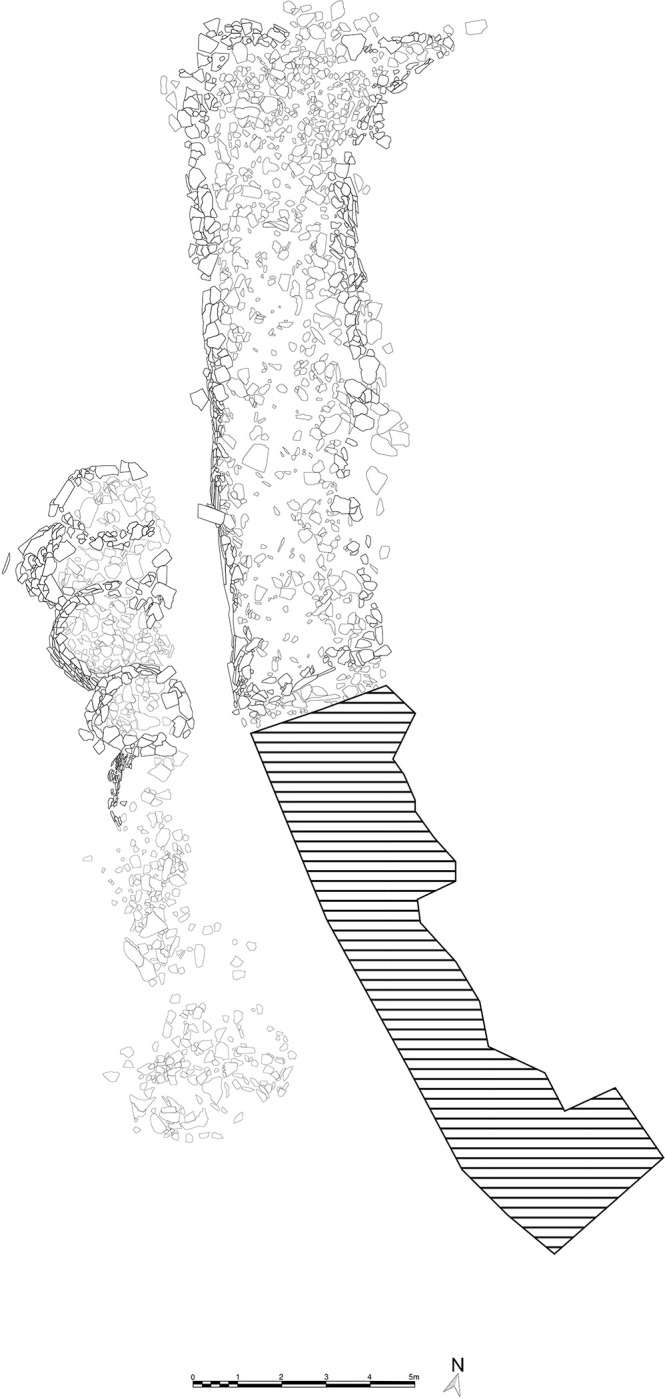
Plan of the base of mustatil IDIHA-F-0011081 and associated feature IDIHA-F-0011149. Hatching indicates unexcavated area.

**Fig 5:**
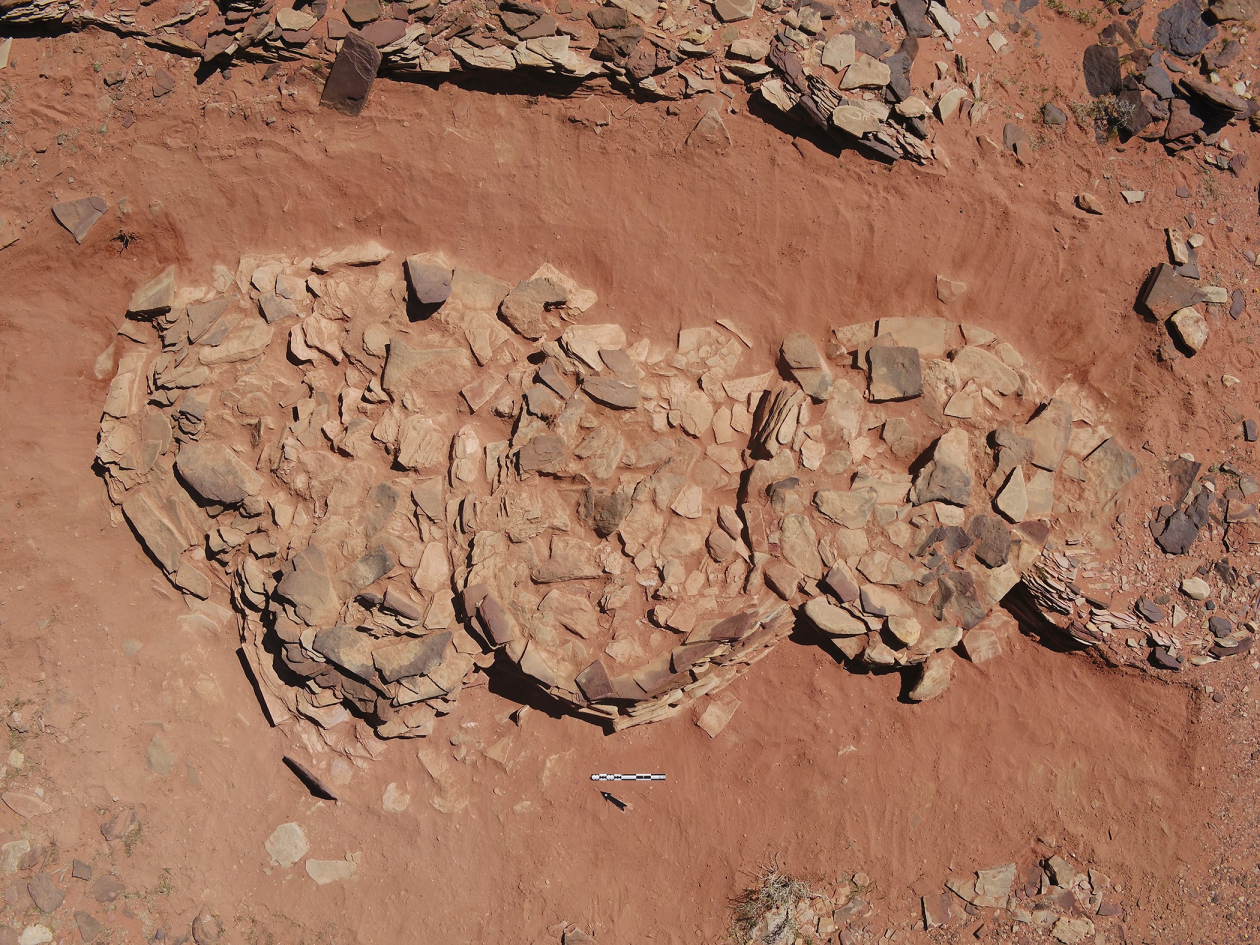
Detail of north-west (preserved) interlocking cells of associated feature IDIHA-F-0011149.

**Fig 6:**
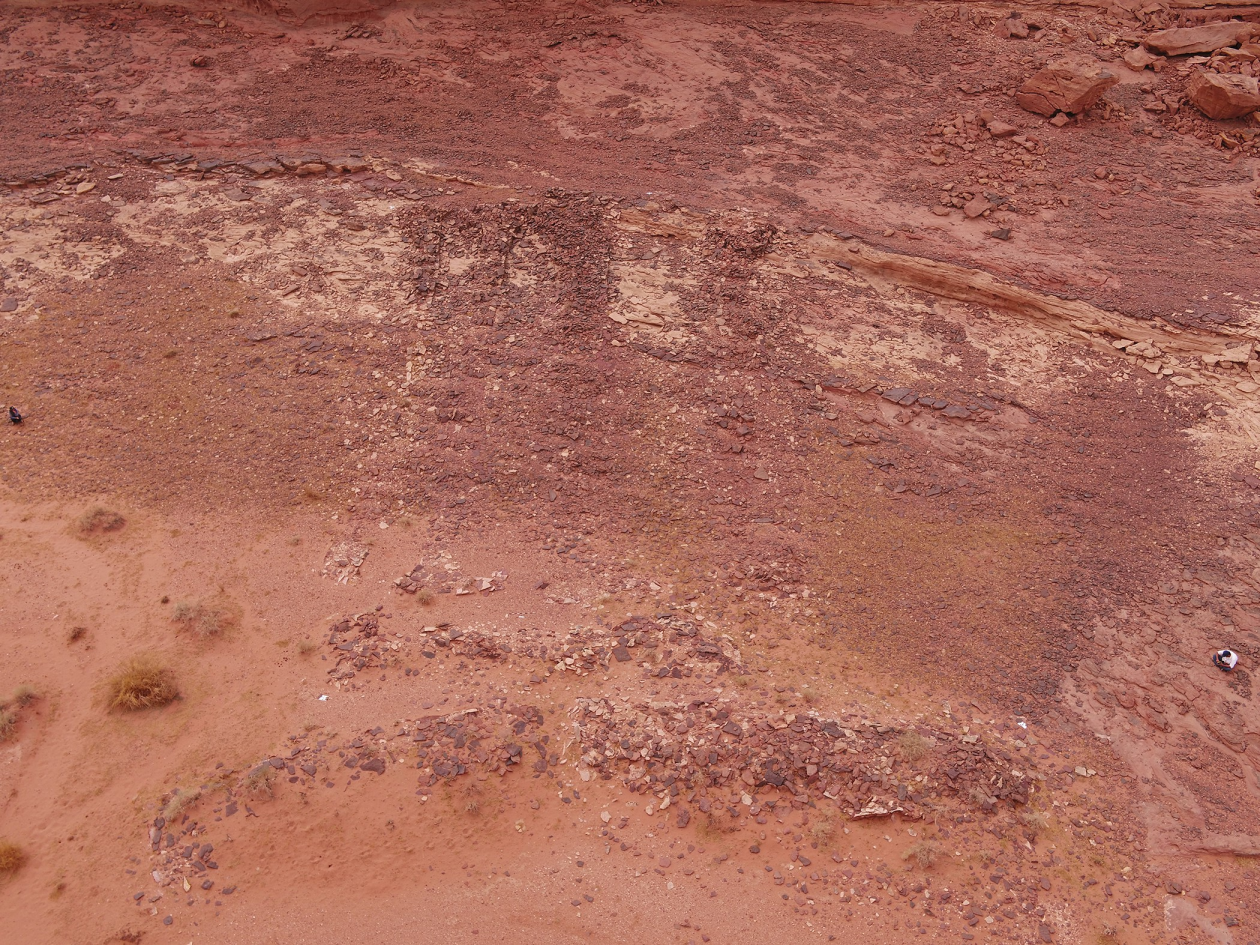
The four I-type platforms located west of IDIHA-F-0011081, photo orientated west.

West of the base, exterior to the mustatil, is a mustatil associated feature (IDIHA-F-0011149), which consists of nine, infilled interlocking cells (Figs 4 and 5). Unfortunately, the southern cells and base have been badly damaged by a wash-gully and as such were not excavated. Further to the west are four large I-type platforms (Fig 6). These features are positioned on the lower slope of a yardang and range up to 30m in length. Like the mustatil, these features are orientated southwest-northeast.

In addition to these larger structures, a small sub-rectangular cist (IDIHA-F-0011213) was discovered immediately to the north of the mustatil, under the rock overhang. Whilst to south, a small, stone-lined circular hearth (IDIHA-F-0011091) was also identified. No occupational evidence could be discerned at the site or within its immediate vicinity, with the nearest, visible settlement (undated) located 4km to the south.

## Methods

### AAKSA project excavation and survey methodologies

Site IDIHA-0008222 was initially identified during the AAKSA project’s intensive remote sensing survey (ca. 19,181 km^2^) of AlUla County in 2018. The site was subsequently photographed from the air by helicopter. The purpose of low-level aerial reconnaissance (helicopter photography) is to rapidly identify, verify and photographically record in higher detail hundreds of sites over a broad geographical area, as well as to identify sites and features not visible in the satellite survey. Based on these photographs, site IDIHA-0008222 was chosen for intensive ground survey. In October 2019, the AAKSA team accessed the site by 4x4 vehicles. All visible features were recorded and pedestrian transects were undertaken around these features and at various points across the site. On site recording included; detailed photography, drone-based and terrestrial photogrammetry along with written notes. These notes included information such as feature/typological form, construction technique and materials, geometry, preservation, as well as measurements of the x, y and z axes. Based on this analysis the site was chosen for excavation. For the excavation component, single context recording was utilised. This included context sheets and diary recording for each excavated feature and context. Recorded details included information regarding sediment colour (measured against a Munsell chart) and consistency; depth and extent; spatial (stratigraphic) relationships; bioturbation/disturbance; as well as sketches of each context, and any other facets deemed of note. Contexts were excavated stratigraphically. All features were excavated by hand, with all sediment sieved through a 2 mm or 5 mm mesh. A 100% collection methodology was utilised. All data was then uploaded to an online relational database based off the ARCHES platform, at both a site (Catalogue: IDIHA-*) and feature (Catalogue: IDIHA-F-*) level. Due to the nature of the ARCHES platform there is no numerical link between site and feature identifiers. The IDIHA database is a customisation of the Getty Conservation Institute’s ARCHES platform by the *Endangered Archaeology in the Middle East and North Africa* (EAMENA) project, University of Oxford, further modified to RCU’s requirements. The AAKSA team was granted permission (permit) to work at this site by the Royal Commission for AlUla.

### Methodology for the analysis of the faunal assemblage

Identification is based on osteological morphology by means of comparative osteological data-sets (Museum of Natural History of Geneva (MNHG) and local specimens), metrical data and specialized literature [8, 9; for sheep and goat determination]; [10–12; for cattle]. When it was not possible to discriminate between domestic and wild taxa, the term *Bos* sp. is used for remains that may belong to *Bos taurus* or *Bos primigenius*. The term undetermined caprines is used for undistinguished *Capra hircus*, *Ovis aries* and *Capra ibex nubiana*, with the term small ruminants used when gazelle could not be excluded. Gazelle identification is based on morphology after [13, 14]; metrics after [15–17]. Measurements are expressed in mm and were performed by the same author following [18] standards. Linear measurements are taken with a sliding caliper (accuracy 0.1 mm), curves were measured with a measuring tape (to the nearest mm). Sex and age determination of horns are based on metrical data, namely basal measurements, compared with specimens in the MNHG and relevant literature.

Age estimation of teeth is based on the eruption and wear of maxillary teeth. Since occlusion connects maxillary to mandibular teeth, the wear stages developed by Grant [19] were adapted for recording maxillary teeth wear, in combination with crown height measurements. Data were then compared to those of 16 cattle specimens of known age at death from the MNHG. Dental wear is related to diet and may be heavier in sandy environments. As such, the examined teeth may present a more pronounced wear, but dental eruption remains a key indicator. Specimens are only attributed to broad age categories.

Estimation of the minimum numbers was performed directly on the material, based on morphology, metrics, fragmentation and lateralisation, and refined by age data. Moreover, in light of the disturbed stratigraphic context of some remains, the MNI estimation has been made across the whole assemblage, rather than by phase.

The terminology adopted here is bucranium and aigicranium for skulls respectively of cattle and goat without mandibles (bucranium used when referring to both); horned frontal for the portion including only the frontal bones with the horns.

### Radiocarbon methodology

Radiocarbon samples were sent to the Australian National University Radiocarbon Laboratory and the Centre for Applied Isotope Studies, University of Georgia for dating. This material included bone, coprolite, charcoal, horn and tooth dentine. The coprolite sample dissolved and did not survive pretreatment. Bone samples SK0002, IB0022, IB0015 and #103 all failed the chemical pretreatment and were not dated. One charcoal sample #0084 also failed in pretreatment. Charcoal sample #0086 was treated with a standard ABA technique, 1M HCl (30 min 70C):1M NaOH (1 hr 70C):1M HCl (30 min 70C), with multiple ultrapure water rinses between. The keratin from horn samples (#0034, #0100, #0037, #0039, #0042, #0043) were pretreated with a modified ABA, 0.5M HCl (1 hour):0.1M NaOH (30 min):0.5M HCl (1 hour), with multiple ultrapure water rinses between. The tooth dentine (#0102 –SANU64603) and bone sample (SK0003) were both pretreated with standard bone protocols, 0.5M HCl (2 hours, room temperature):2M HCl (overnight, 5C): 0.1M NaOH (30 min, room temperature): HCl pH3 (0.001M, 70C, 20 hours):ultrafiltration (Sartrorius Vivaspin Turbo). All samples were then converted to CO_2_ in the presence of CuO and silver. The CO_2_ was then converted to graphite using H_2_ and iron powder. The graphite samples were then measured on the Single Stage Accelerator Mass Spectrometer at the Australian National University. Samples were normalized using Oxalic Acid I, background subtracted with ^14^C free material (coal and bone). Results are presented as F^14^C and radiocarbon age [20, 21] (Fig 20). Radiocarbon results were converted to calendar age and modelled in OxCal v.4.4.4 and the IntCal20 atmospheric curve [20, 21]. Each date was subject to a general outlier model with an outlier probability of 5% [21].

### Methodology for the analysis of human remains

Due to the sensitivity of working with human remains a number of protocols were put in place. Firstly, the remains (SK003) were excavated by a qualified forensic anthropologist. These were excavated stratigraphically and were kept *in-situ* for as long as possible. Once cleared of sediment the remains were carefully lifted, following the appropriate field documentation and recording (drawing, photographs, measurements and registration). To ensure the recovery of even the smallest of bones, the entire contents of the cist was sieved through a 2 mm mesh. Initial processing and curation of the skeletal remains meant that all bones were labelled individually with a prefix designating the specific finds local, with these elements wrapped in acid-free paper. Anthropological analyses of the human skeletal remains were firstly, aimed toward ascertaining the minimum number of individuals (MNI). Secondly, to outline biological identifiers which inform the demographic of the individual(s) within. The assessment of the MNI was based on atypical replication of skeletal elements and divergent demographic data (e.g., skeletal age) between individuals. Traditional metric anthropological assessments of skeletal sex were based on published standards [22] derived from a medieval east Anatolian population, the most geographically and temporally consistent with the recovered remains. Age estimation was based on complete fusion of all recovered skeletal elements taking into consideration degenerative changes present throughout the skeleton. An assessment of skeletal pathology and trauma was also conducted to provide insight into the lifestyle and health status of the individual recovered.

## Results

### Stratigraphic overview of Mustatil IDIHA-F-0011081: The HEAD

**Fig 7:**
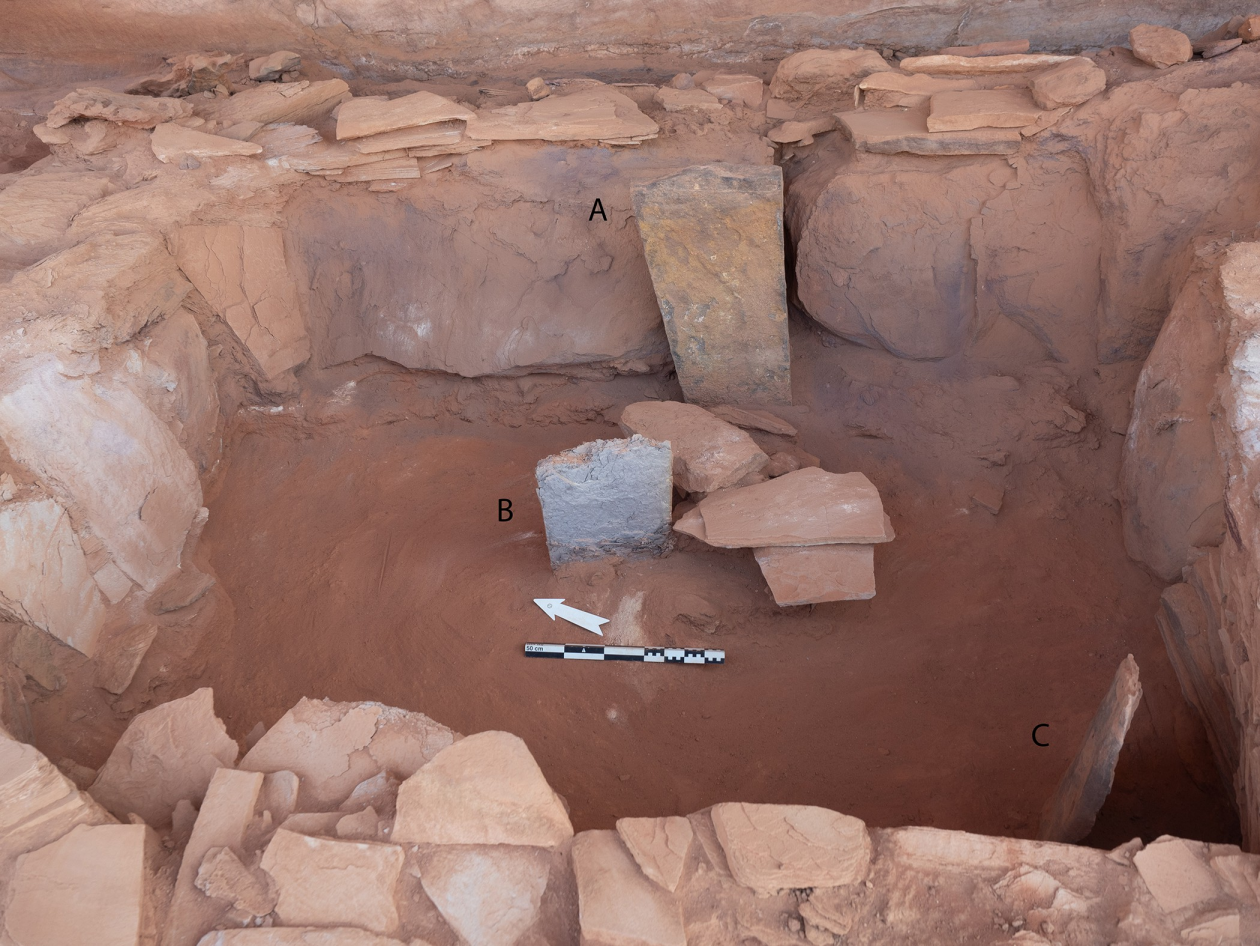
The main (central) chamber of mustatil IDIHA-F-0011081 with three up-right stones (A-C). Flat stones in centre of image acted as support for primary up-right stone in the rear. The blocked doorway is visible in the left of the photo.

**Fig 8:**
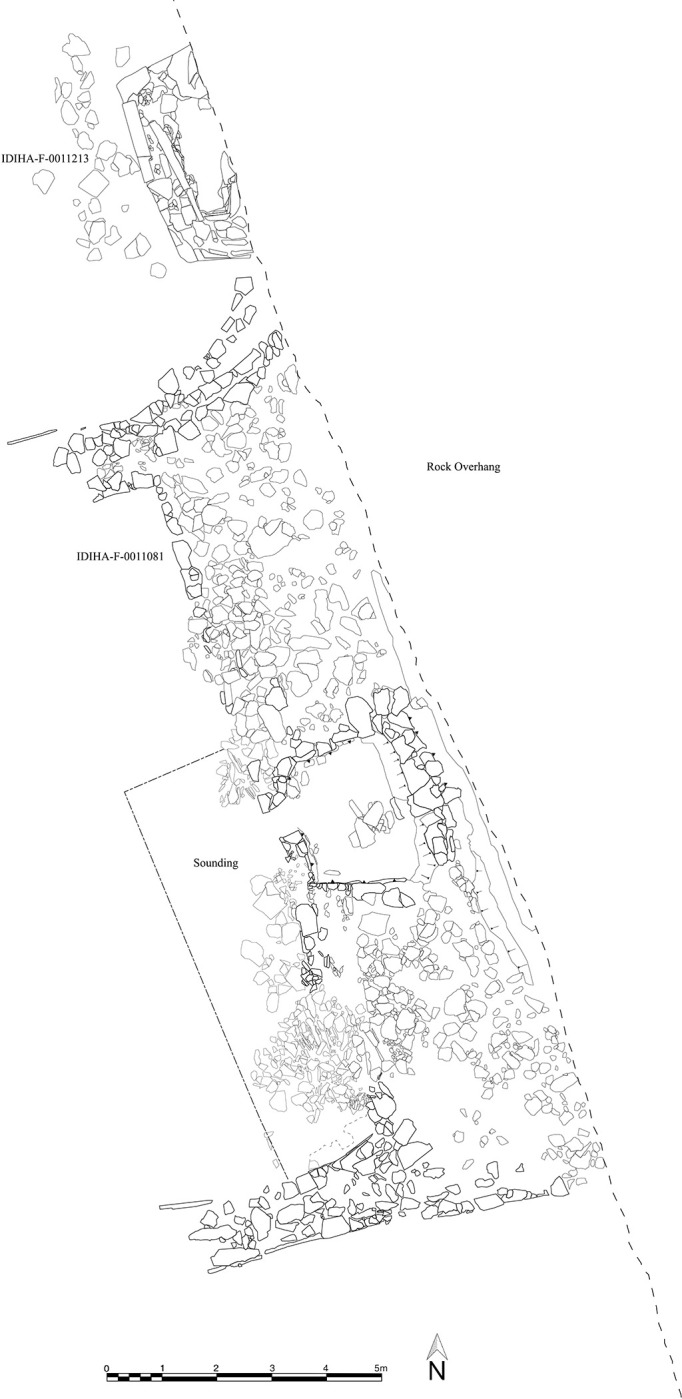
Plan of the head of mustatil IDIHA-F-0011081, with the cist IDIHA-F-0011213 located to the north.

Excavations in the head of the mustatil revealed two chambers. The first is a long linear channel positioned in the east (rear) of the head, against the rock overhang and formed from the underlying bedrock, measuring 0.6 x 0.9 x 5.5m. Immediately to the west, is the second chamber (Figs 7 and 8). Characterised by a trapezoidal shape, this semi-subterranean chamber is located in the centre of the platform and can be considered the main chamber. This chamber measures 3.10 x 2.80 x 2.5 x 2.85m and is constructed from a mixture of unworked slabs of sandstone desert pavement and the underlying bedrock. The eastern wall of the chamber is characterised by gently sloping, carved bedrock. On top of which sat a narrow wall, two-three courses high and one stone wide. The northern and southern walls of the chamber are characterised by a mixture of bedrock and vertically set sandstone slabs. Whilst the western wall is composed entirely of courses of thin unworked sandstone. In the western wall was a small, blocked doorway measuring 0.56 x 0.46 m and opened to the courtyard.

#### The main (central) chamber

Positioned in the centre of the chamber against the eastern wall was a large (primary) ashlar up-right stone (A), measuring 0.8 x 0.43 x 0.1m. A second up-right stone (B) was located to the west and slightly north of it, and a third, smaller, up-right stone (C) was positioned in the southwest corner of the chamber (Fig 7). Excavations within this part of the feature suggest four phases of activity (Phases 1–4). Two of these phases (Phases 3 and 4) were divided into sub-phases during post-excavation analysis. Due to the homogenous nature of the deposit within the chamber, stratigraphic changes and the association between finds was not always clearly discernible during the excavation process. However, post-excavation analysis of the finds suggested that these phases could be further refined, as a means of ensuring greater stratigraphic differentiation across the corpus. As such these sub-phases reflect this stratigraphic ambiguity. Analysis of the faunal remains from this chamber revealed 209 NISP.

#### Phase 1

**Fig 9:**
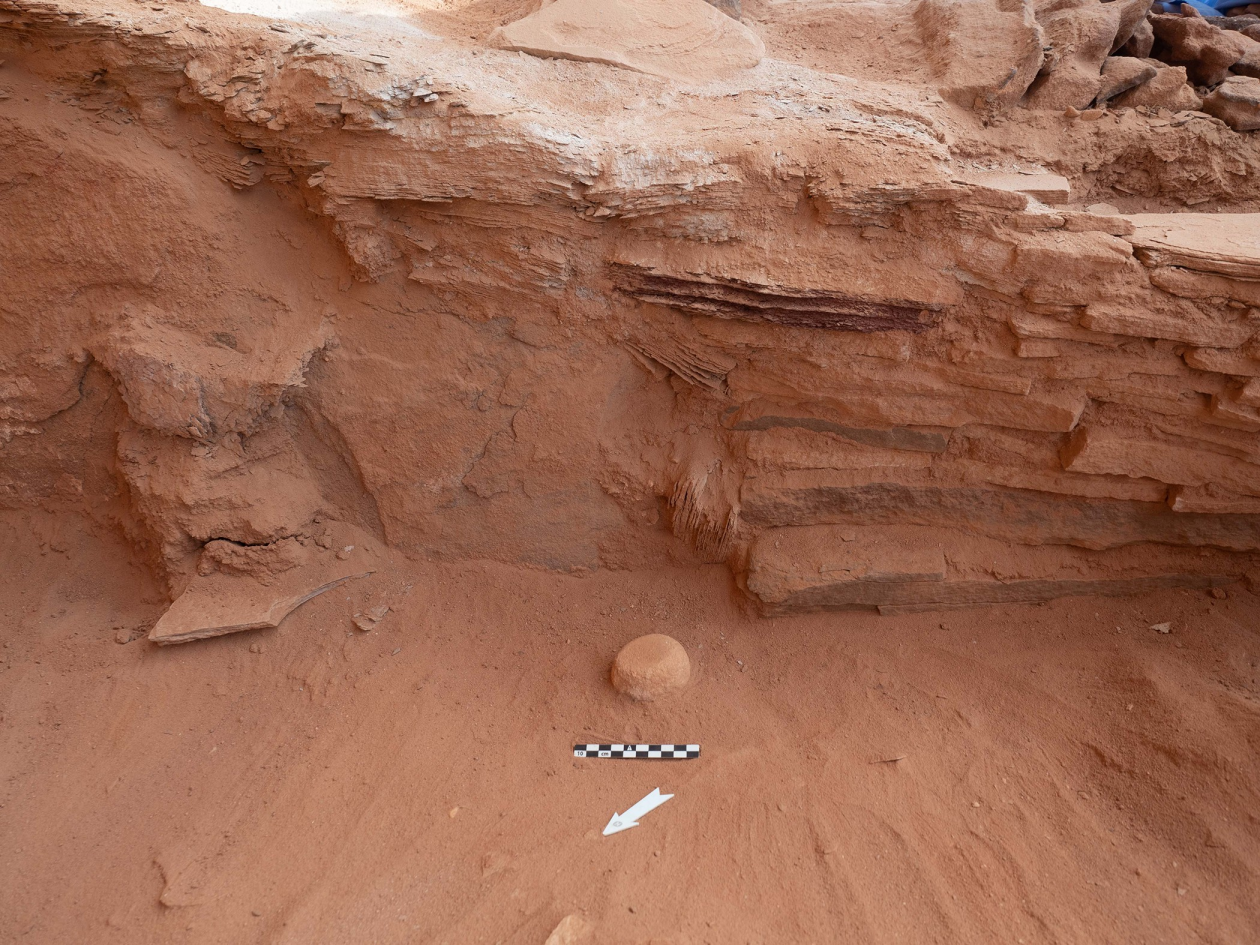
Transverse grooved hammer stone found upside down and in situ in Phase 1.

Phase 1 represents the initial construction episode. In addition to the construction of the chamber, a series of unworked stones were positioned west of the up-right stone (A). These stones functioned as a structural support for stones A and B, located in the east of the chamber. A similar support was also found for stone C in the southwest corner. These support stones were sealed by 20-30cm of sterile, dark red-to-purple sand and decayed bedrock. As no foundation cuts could be identified for the up-right stones and packing (with the exception of stone C, which was chocked), it appears that this horizon was most probably redeposited natural from a shallow excavation to level the area under the overhang. Above the stone packing and redeposited natural was a 5-10cm layer of orangey-red sand. Situated within this horizon, in front of and to the south of stone A were two roughly circular hearths and poorly preserved faunal remains (including teeth and bone fragments). These hearths were ephemeral and could not be sampled, suggesting they were exposed to the elements. The open-air nature of the chamber is also indicated by the fact that no roofing material was identified during the course of excavation, whilst the recovery of animal coprolites, may imply a brief lacuna between Phases 1 and 2, and/or that the chamber was not cleaned prior to Phase 2. Several ground stone tools and chipped stone artefacts were also recovered (Fig 9).

The earliest five identified faunal remains pertain to Phase 1 (a goat petrous bone, a caprine tooth fragment and cattle teeth).

#### Phase 2

**Fig 10:**
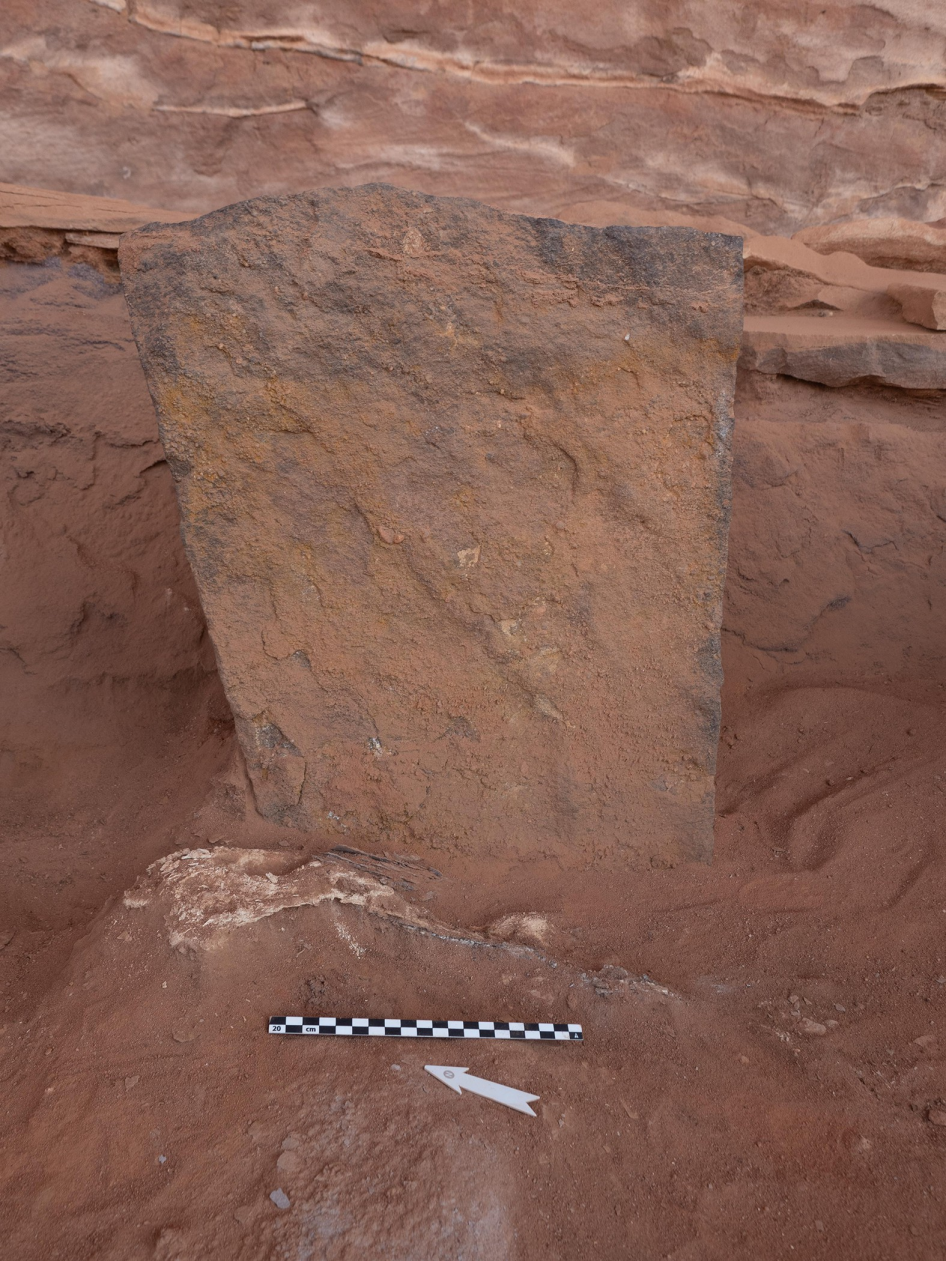
Horned frontal of a male domestic goat (#N01) found in Phase 2.

The earliest context of Phase 2 was located above the orangey-red sand of Phase 1 and is best described as a partial stone surface. Constructed from unworked sandstone slabs, this surface was located immediately to the west of stone A and did not extend over the entirety of the chamber. Two (preserved) remains pertain to Phase 2, this includes a horned frontal of a male domestic goat (#N01), placed directly in front of stone A, representing one of the two clearly original, intentional deposition activities found within the chamber (Fig 10). Unfortunately, the faunal remains of this episode were poorly preserved, with the vast majority disintegrating upon excavation. The faunal remains of Phase 2 were closely associated and appear to have been deposited as a single event, sealed by 15-20cm of sand and stone, which marks the interface between Phases 2 and 3.

#### Phase 3

**Fig 11:**
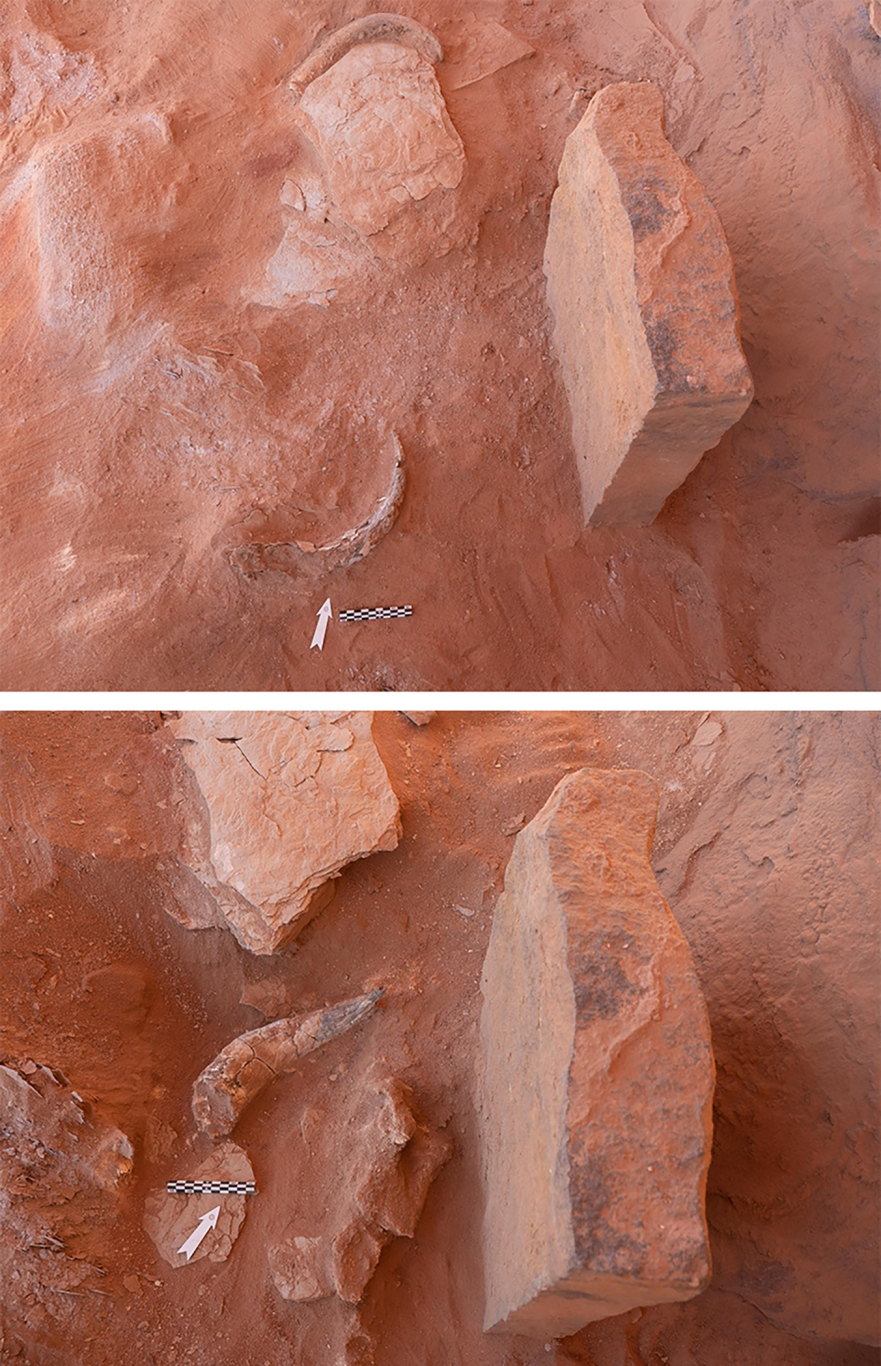
Cattle horns found in association with the betyl in Phase 3 (10 cm depth between the two images). Top image (#0034 –top) and (#0039 –bottom). Bottom image (#0037).

Like the preceding phase, a sequence of cranial elements and horns were located in the east of the chamber, around stone A (Fig 11). However, the faunal remains of Phase 3, particularly the horns, were better preserved. Defining stratigraphic changes within this phase, and indeed the entire sequence of the chamber, was extremely difficult as the matrix was characterised by a homogenous deposit of sand. Nonetheless, two distinct offering events could be identified in Phase 3, Phases 3A and 3B. The earliest sub-phase (3A) was positioned approximately 15cm above the faunal remains of Phase 2 and consisted of a cattle sheath (#0037) and a bovine sheath (#0035) positioned flat but with different orientations around stone A. These remains were sealed by a 10cm layer of sand, the boundary between Phases 3A and 3B. The doorway to the chamber was also blocked during this sub-phase, with several lithics of undiagnostic debitage production found within this blocking.

One-hundred and fourteen remains were recovered from Phase 3B, including teeth, neurocranium fragments and horns. These were not placed on a single surface and were mixed. Bovines, caprines and gazelle (#0044) are represented in this horizon, with a significant number of horns including: two goat horn sheaths (#0036 and #0042, the latter found southeast of stone A), three bovine horn sheaths (#0034 and #0039, lying flat with different orientations on the same level together, 40 cm from stone A, and #0045). A *Bos* sp. maxillary was found upside down, lying on a collapsed oblique stone. This phase was then sealed by 15-20cm of sand and rock, marking the interface between Phases 3 and 4. This sealing may not have been contemporary with the initial presence of these remains, as suggested by taphonomy and the radiocarbon data. Similarities between sheaths #0037 and #0039 belonging to distinct phases (Phases 3A and 3B), and distinct taphonomy on sheaths from the same context (cattle sheaths #0037 and #0039, but also as the goat sheaths #0036 and #0042) speak to the complexity within Phase 3 overall which may be a result of its exposure prior to Phase 4. Significantly, more stone was found in association with this sealing event than the preceding between Phases 2 and 3. These stones were incredibly friable and laminated on contact, with this stony rubble both flat lying and diving at angles.

#### Phase 4

**Fig 12:**
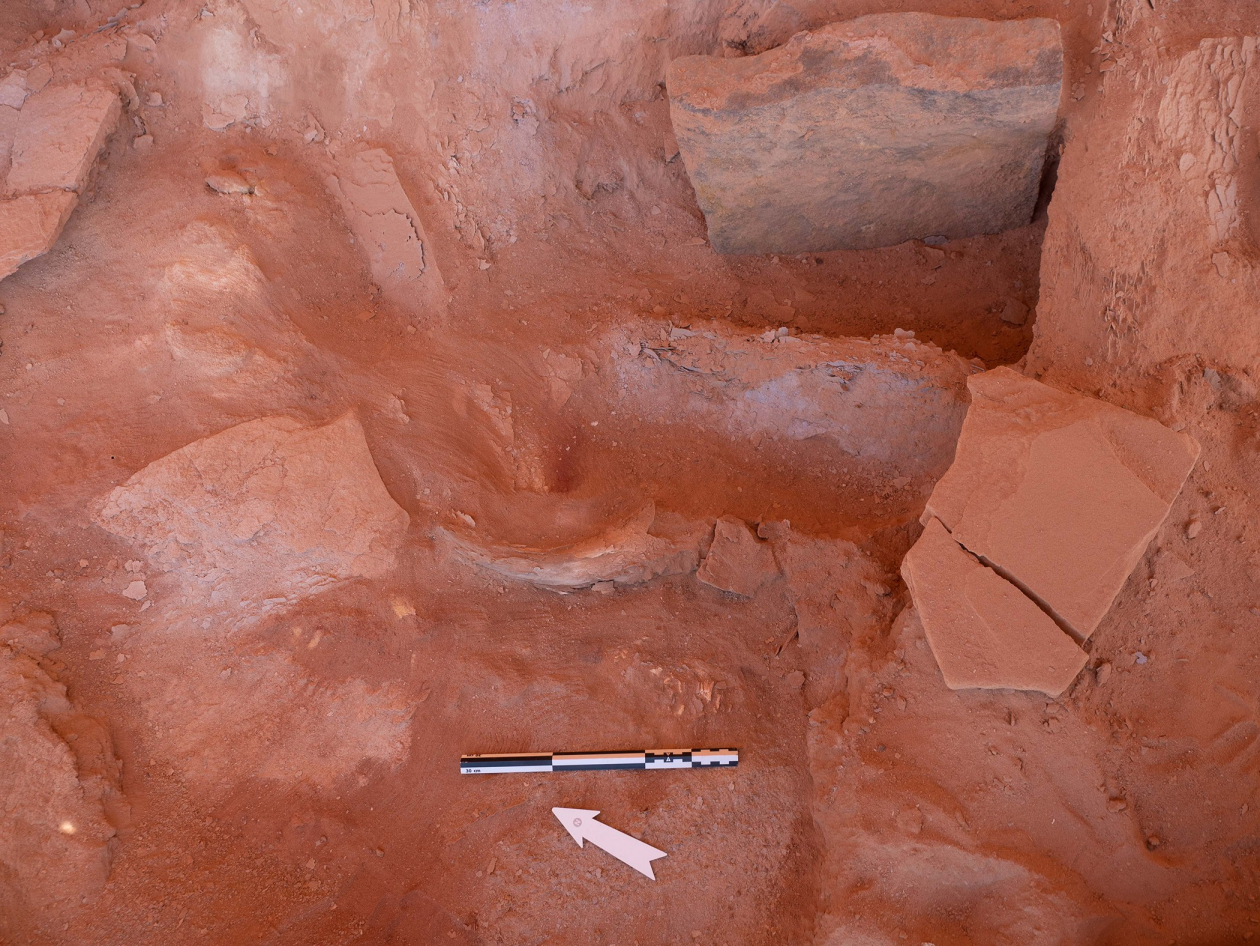
Bos sp. horn (#0032) recovered from Phase 4A, note the positioning in relation to up-right stone A.

**Fig 13:**
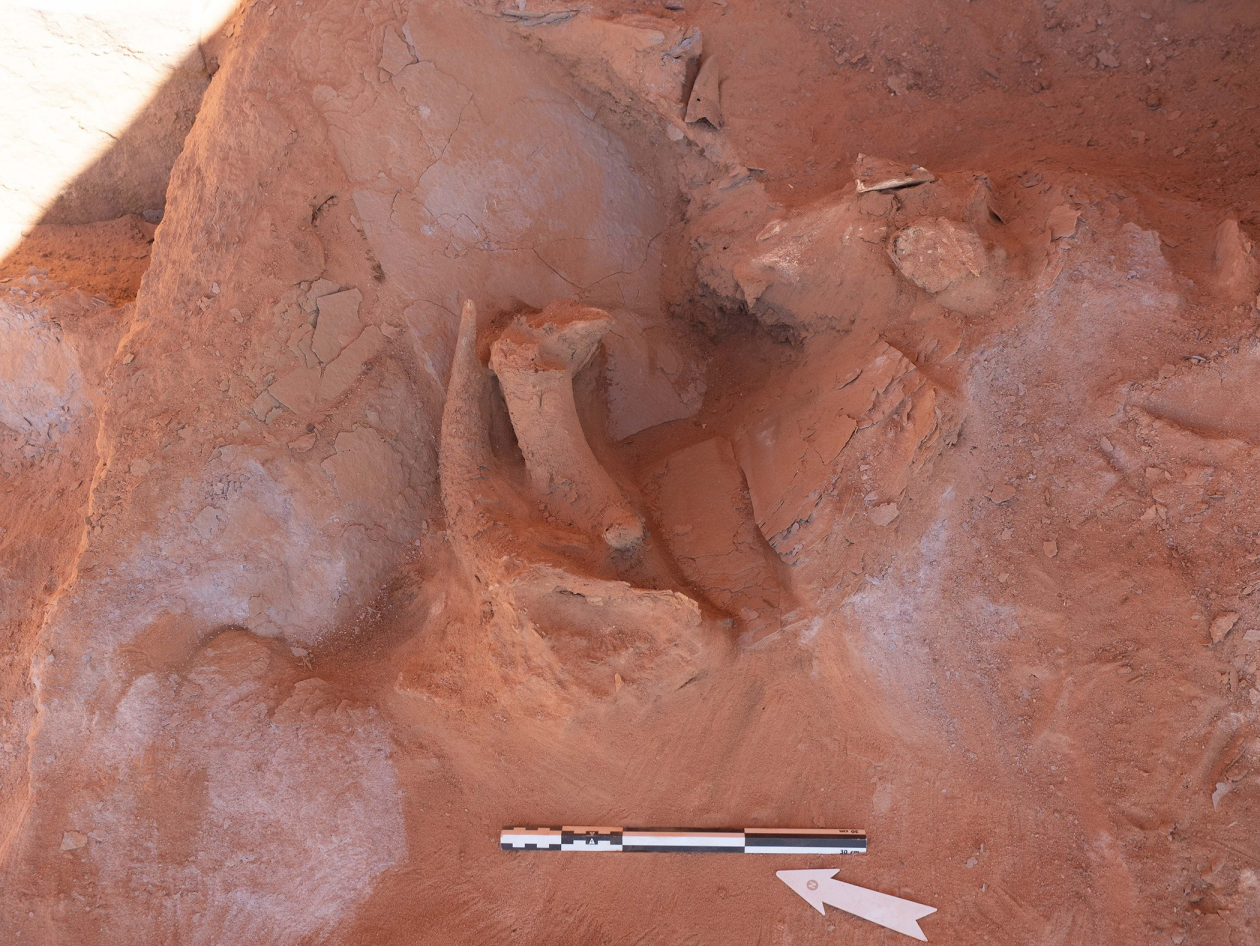
Horns found on a collapsed “bench” in Phase 4B. Left to right, large cattle horns/sheaths (#0043) and (#0033), goat horn sheath (#0047), goat (#0041), and cattle horn (#0040).

**Fig 14:**
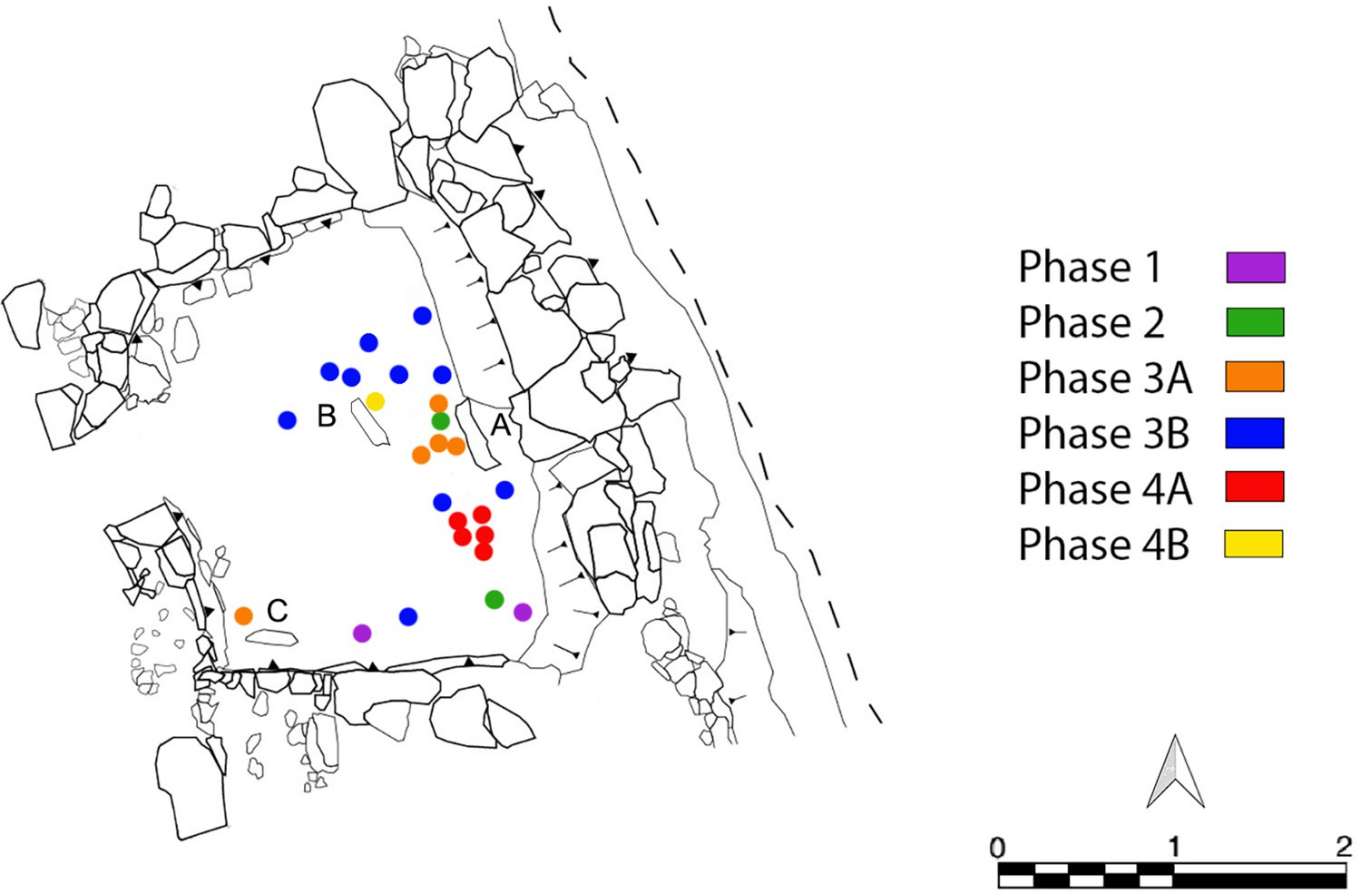
Plan showing location of faunal elements and other artefacts during the four phases of use.

Phase 4 represents the terminal phase. As with Phase 3, at least two layers of faunal remains could be discerned within this horizon (Fig 14). The first (Phase 4A), positioned directly on top of the final Phase 3 sealing event, consisted of a *Bos* sp. horn sheath (#0032) located 35cm to the north-west of stone A (Fig 12). Loose cattle and caprine teeth were also recovered from this horizon (but were not preserved and are not included in the counts. This horn sheath (#0032) was recovered less than 5cm below the modern surface and is extraordinarily well-preserved. Interestingly, this horn was found in association with a considerable amount of animal bone, which in comparison to Phase 3 was poorly preserved and could not be analysed. A further well-defined and well-preserved deposit of five horns and cranial elements was found to the south of stone A on what can be described as a stone “bench” (Phase 4B; Fig 13). This cluster includes a male goat horn sheath (#0041), a cattle horn (#0033), a cattle horn sheath (#0043), two *Bos* sp. horn fragments, cattle maxillary teeth, including an incisor, and petrous bones. These elements represent the second *in situ* intentional deposition activity (additional to that in Phase 2). The cattle horns were arranged parallel in opposite orientation (Fig 12), This “bench” was crudely made of laminating sandstone slabs and had collapsed. Due to the positioning of the collapse, it would appear that this feature may have been constructed over a void, this is further supported the fact that conjoins between horns were found between Phases 3B and 4B, specifically goat horn sheath #0041 and its tip #0036 found in Phase 3B, immediately below the bench. Further bovine maxillary teeth and a cattle petrous bone also belong to this phase. Preservation appears to have been aided by the fact that the horns were encased in a reddish-brown clay, giving the horns their colour. The clay and the underlying deposit differed considerably from the surrounding soil, indicating the horns were deliberately sealed or encased, immediately after deposition. Above this clay was a 5cm layer of natural, wind-blown sediment which represents topsoil or the modern surface-level of the chamber (Fig 14).

#### The secondary (rear) chamber

**Fig 15:**
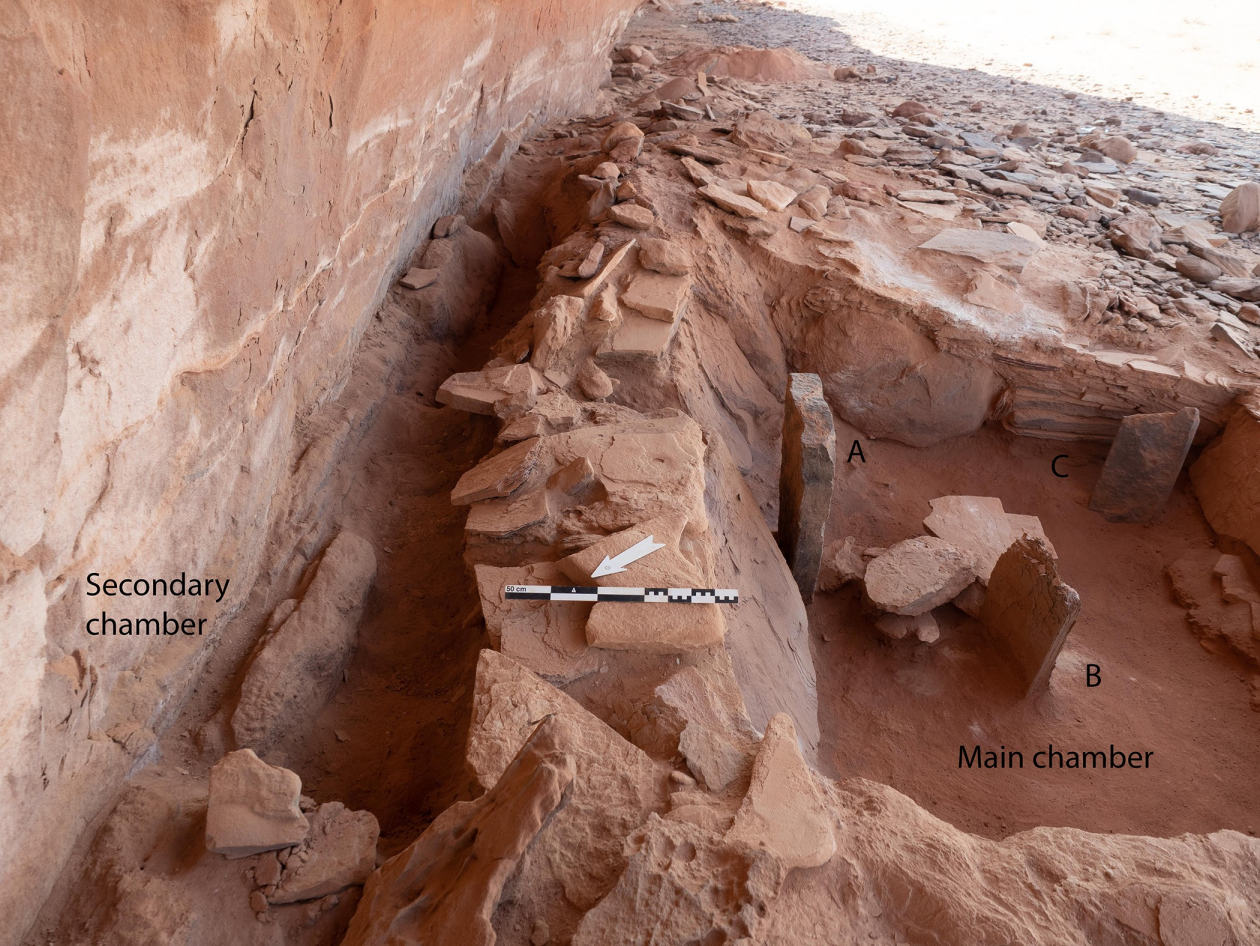
Spatial relationships between the main and secondary chamber.

The secondary (rear) chamber was located in the east, against the rock overhang (Fig 15). Excavations in this part of the structure revealed a homogenous deposit of sand, within this a small collection of faunal elements, including a goat horn and teeth were recovered. Unfortunately, as both chambers were architecturally/spatially separated, a clear stratigraphic association between the main and secondary central chambers could not be determined.

The finds in the rear chamber include 38 NISP, in the lower portion of the chamber (21 NISP), bovine and caprine cranial fragments and loose teeth, a goat frontal with horn core base and a bovine sheath fragment were recovered. The upper context revealed the remainder of the corpus specifically cattle maxillary teeth and *Bos* sp. cranial fragments.

The presence of faunal remains in the rear chamber may suggest that some of these elements were originally deposited there, suggesting two distinct areas of ritual focus. These elements, loose teeth and nasals, are components that easily fall from the skull after decomposition, and as such may have been positioned (exposed) on the rear (eastern) wall of the main chamber, overlooking both the main and secondary chambers.

### Description of the faunal remains from Mustatil IDIHA-F-0011081

Two hundred and sixty faunal remains were recovered inside the two chambers of mustatil IDIHA-F-0011081, corresponding to a weight of 3,311.9g (excluding non-recovered finds). The number of identified specimens (NISP) is 246, corresponding to 3,201.9g.

The faunal assemblage comprises 89 remains of domestic cattle (*Bos taurus*), 128 remains of *Bos* sp., five remains of domestic goat (*Capra hircus*), 19 remains of undetermined caprines, one remain of gazelle (*Gazella* sp.), and five remains of undetermined small ruminants (Table 1). All the identified remains are elements of the skull (Table 1). The assemblage includes parts of the cranial box, comprising four petrous bones, and parts of the face, encompassing maxillary teeth fragments (207 remains). As for horns, the horn core (bone nucleus) is present with seven remains, the horn sheath (keratin shell that covers the bone nucleus) with 27 remains, and less frequently the whole horn (bone and sheath) with four remains. Mandibles and mandibular teeth are absent, as well as any other anatomical element. The outer layers of keratin are cracked and flaked or have a fibrous aspect. The core is dark and translucent. Three colours are observed: reddish brown, brown and dark grey/black.

**Table 1:** Quantification of identified faunal remains from mustatil IDIHA-F-0011081 (NISP = number of identified specimens).

Taxon	NISP	% NISP	Weight (g)*	% weight	MNI**	Anatomical element per taxon	NISP	MNE**
**Cattle**	89	36.0%	2223.2	69.1%	8	**Cattle**	89	82
						horn***	1	1
						horn core	0	0
						horn sheath	4	4
						cranium	11	4
						upper tooth	73	73
***Bos* sp.**	128	51.8%	810.5	25.3%	0	***Bos* sp.**	128	19
** **						horn	1	2
** **						horn core	6	0
** **						horn sheath	21	2
** **						cranium	13	0
** **						upper tooth	85	15
						tooth fragment	2	0
**Goat**	5	2%	153.3	4.8%	3	**Goat**	5	4
						horn sheath	2	2
						cranium + horn core	1	1
						cranium + horn	1	1
						cranium	1	0
**Ind. caprine**	19	7.7%	15.9	< 1%	0	**Ind. Caprine**	19	6
** **						cranium	1	0
** **						upper tooth	18	6
**Gazelle**	1	< 1%	3.0	< 1%	1	**Gazelle**	1	1
** **						horn	1	1
**Small ruminant**	5	2%	6.0	< 1%	0	**Small ruminant**	5	0
						cranium	4	0
						tooth fragment	1	0
**Total**	**247**	**100%**	**3185.9**	**100%**	**12**	**Total**	**247**	**112**

* Excluding non-preserved specimens

** estimation considering the whole assemblage

*** horn = horn core + sheath.

#### Bovines

Most of the remains belong to bovines: 217 remains, corresponding to 87.8% of the NISP and weighing 3,033.7g (95.2% of the total weight). Eighty-nine remains (36.0% of the total NISP) are attributed to domestic cattle (*Bos taurus*), whereas 128 remains (51.8%) could not be attributed at the species level and are recorded as *Bos* sp. since aurochs could not be excluded (Table 1; cf. [23–26]).

**Fig 16:**
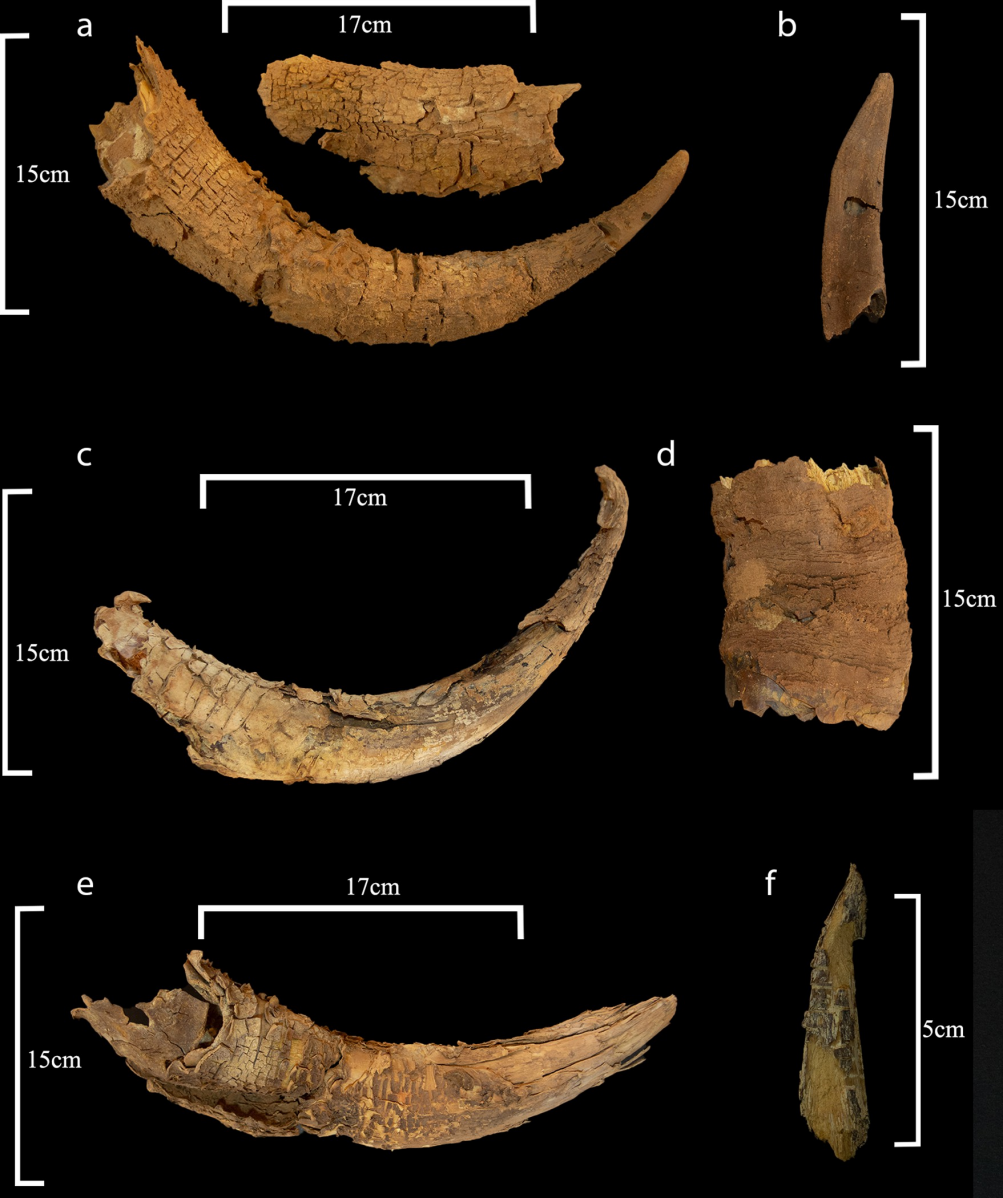
Horns recovered from mustatil IDIHA-F-0011081 –a: Cattle horn (#0043). b: Goat horn (#0036); c: Cattle horn (#0034). d. Cattle horn (#0040). e. Bos sp. horn (#0032). f. Gazelle horn (#044).

All fragments belong to the skull, with both sides represented for all anatomical portions. Namely, 11 cattle and 13 *Bos* sp. remains are part of the neurocranium, maxillary bone, including three cattle petrous bones (altogether 12.4% of the bovine remains); 158 remains are maxillary teeth (73 for cattle), 72.8% of the bovine remains. Cranial boxes are heavily fragmented. Horns represent 15.2% of the bovine remains and include one cattle and one *Bos* sp. horn fragments, four cattle and 21 *Bos* sp. horn sheath fragments, and six *Bos* sp. horn core fragments (Table 1; Fig 16). Twenty-four fragments belong to the basal and mid portion, in three cases only the tip is preserved, and in six cases more than half of the horn. Among the latter, five are attributed to domestic cattle based on morphology. The outer curvature of their sheath is longer than 300mm (Table 2), classified as medium and long horns [27]. As for morphology, #0043 and #0035 develop laterally straight ends pointing upwards with no torsion. #0034 (SANU-65412) is closely comparable but is damaged at the tip. #0033 and #0037 (SANU-66605) are shorter, rise upwards and slightly twist backwards at the tip. Sheath #0032 is damaged and therefore its exact morphology cannot be observed, however, it presents a slightly different development of the tip and is thicker. All of which indicates individuals older than 2 years (outer curvature ranging between 320mm and more than 480 mm; [28]). Horn sheath tips are also well developed, a feature not observed in juveniles [29], sheath rings are not observable [11]. Horn core compactness and surface texture of three remains are at Stage 5–6 [30], thus are considered adults.

**Table 2:** Horn measurements (mm) from mustatil IDIHA-F-0011081 following [18] standards.

Specimen	Length (mm)	Basal circumference (mm)	Maximum diameter (mm)	Minimum diameter (mm)
**#0033 Cattle**	320	210	73	62
**#0034 Cattle**	335			
**#0037 Cattle**		±220	80	
**#0043 Cattle**	480	240	86.5	63.5
**#0032 *Bos* sp.**	360	220	85	
**#0048 *Bos* sp.**			70	
**#0082 *Bos* sp.**			68	
**#0036-#0041 Goat**	230		47.9	
**#0042 Goat**	280		46.7	
**#N01 Goat**	±330			
**#0044 Gazelle**	59.1		11.6	11.2

#0036 and #0041 are matching fragments of the same specimen.

The minimum number of individuals (MNI) is based on teeth (nine permanent upper P2, six left and three right, and two deciduous upper D4, whose wear stage and root shape suggests the animals were close to tooth replacement). The estimate is of eight individuals: two sub-adults around 2.5 years, one young adult around 3–4 years, two adults of 4–9 years and three individuals above 9 years, and possibly over 12 years. However, MNI based on horns suggests five individuals, out of which four to five were domestic cattle, including three to four males. Sex determination is based on horn basal measurements [27, 31]. Considering basal circumferences (210-240mm) and diameters (maximum diameters 70–86.6mm; minimum diameters 62–63.5mm), four cattle specimens (#0033, #0037, #0039 –SANU-66606 and #0043) and two *Bos* sp. (#0048 and #0082) have an oval cross-section and fit male metrical ranges (Table 2) [10, 27, 28, 32]. #0032 *Bos* sp. sheath is also a male, if domestic. Based on petrous bones an MNI of three domesticates is recorded.

The estimated minimum number of elements (MNE) considering the different portions of the skull separately is three horns, six horn sheaths, two maxillaries, five incisor bones, three nasals, one basioccipital, three petrous bones, two frontals, one temporal and 88 teeth.

#### Caprine

Caprine remains are 24 (9.7% of the total NISP; Table 1) and weigh 169.2 g (5.3% of the total). They include five remains of domestic goat *Capra hircus* (2% of the total; 153.3g; excluding the partial goat skull #N01 which disintegrated during excavation); 19 remains of undetermined caprines, either domestic goat or sheep *Ovis aries* or wild *C*. *ibex nubiana* (7.7%; 15.9g); and five remains of small ruminants (2%; 6.0g).

Goat remains include a left petrous bone, two horn sheaths with typically domestic twisting (#0042, #0036-#0041; see [33]; Fig 16B), a fragmented left frontal bone with a portion of the horn core (#0052) and a bilateral frontal bone with both horns (cores and sheaths; #N01; Fig 10). Undetermined caprine remains consist of a left maxillary bone with P4 and M1, six maxillary teeth (five molars, one premolar), one decidual D4 and 11 upper tooth fragments. Small ruminants are represented by a single tooth fragment and four neurocranium fragments.

The goat horns correspond to both adults or sub-adults when compared to specimens in the MNHG. The goat petrous bone is not a juvenile [34]. The wear and root shape of the caprine D4 indicates the animal was close to tooth replacement, around 1.5 years, this estimate is based on the observation of 14 comparative skulls of known age in the MHNG collection. The maxillary belongs to an adult. No predominant side was recorded. Sex determination based on horn metrics and frontal protuberance based on comparisons with the MHNG collection, indicate all specimens are male (Table 2). The MNI estimate is three males: one adult, one sub-adult and one adult/sub-adult. Considering the different cranial portions, the MNE includes two horns, one horn core, two sheaths, three frontals, one petrous bone, one maxillary and six teeth.

#### Gazell

Gazelle is represented by a single horn tip (#0044; maximum length 59.1mm; Fig 16F). The preserved portion is straight, slightly curved or damaged at the tip, the non-basal cross-section is round despite damage. The sheath is present but only some fragments are still attached to the horn core, cracked, with no annulations. The length suggests an animal older than 5 months [15]. The slenderness and shape of the horn are consistent with a female. Different species and subspecies of gazelle are distributed in the area [13, 14, 17, 35–37]. These are the mountain gazelle (*Gazella gazella*), the Arabian gazelle (*G*. *arabica*), the dorcas gazelle (*G*. *dorcas* and *G*. *d*. *saudiya*), the goitred gazelle (*G*. *subgutturosa*), and the marica gazelle (*G*. *marica*). In mountain gazelles, the female horn basal diameter is 9–11 mm [15, 16]. Horn #0044 is larger (non-basal measurements 11.6 mm and 11.2 mm), so mountain gazelle may be excluded. Nevertheless, the diagnostic basal portion is missing and the specimen is damaged, and is insufficient for precise identification. Based on the available data the MNI is one female with an MNE of one horn.

#### Anthropic marks

**Fig 17:**
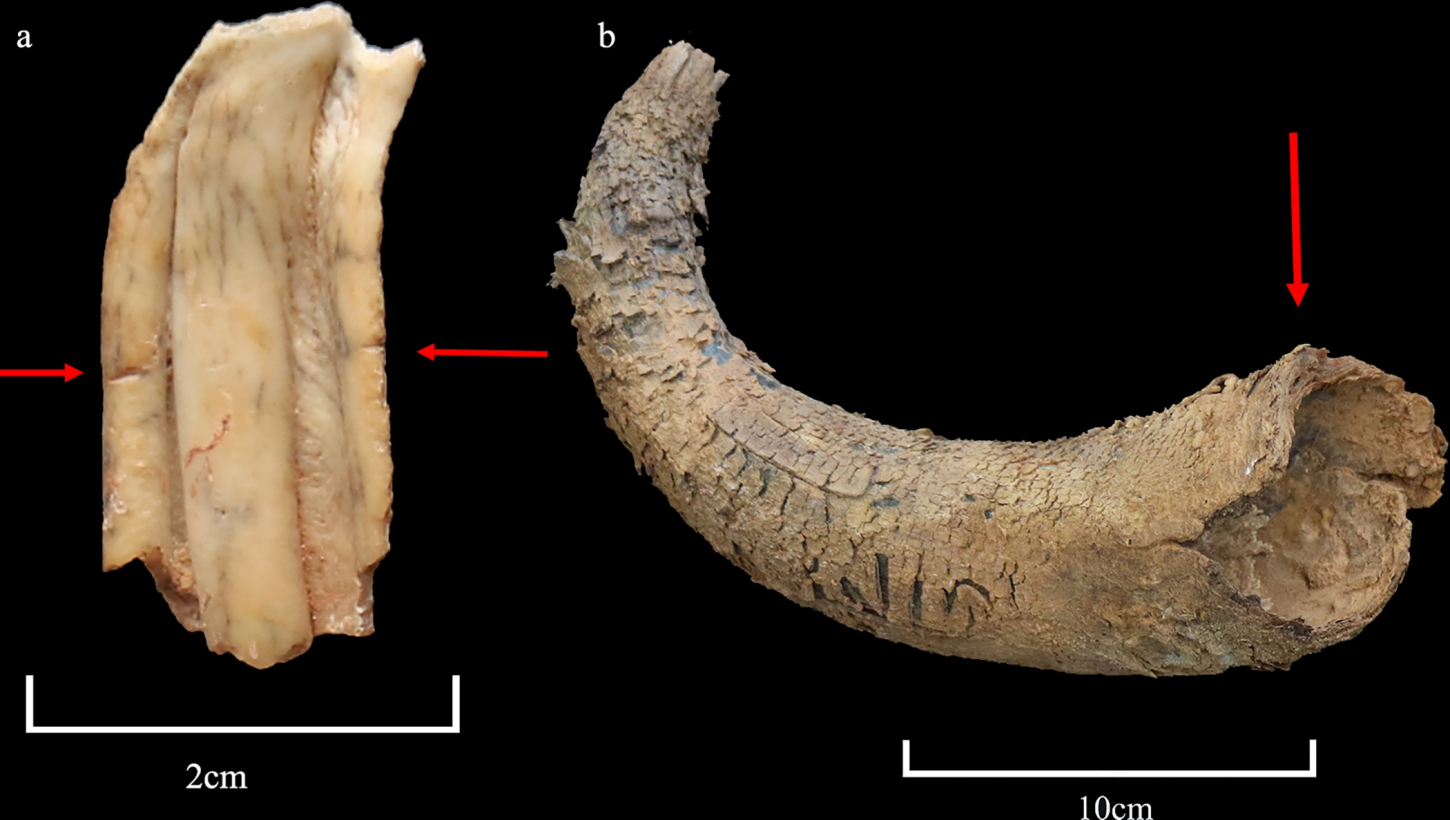
Detail of anthropic marks. a. Cattle left upper molar #0056 presenting three aligned cut marks on its buccal face. b. Chop mark visible on the basal portion of the cattle horn #0033.

**Fig 18:**
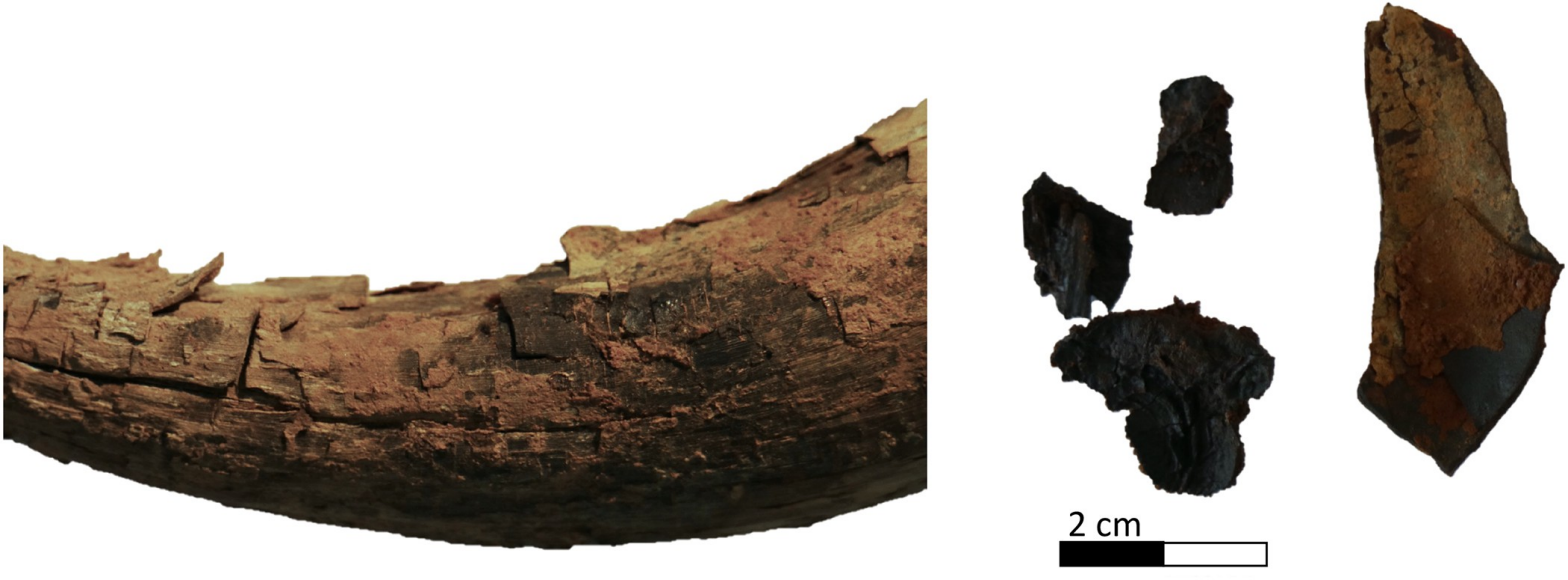
Sheath #0034 with a burnt area and burnt flakes from the inner layers.

Preservation hampers the observation of anthropic marks. Nevertheless, one cattle upper left molar (#0056) presents three aligned cut marks on its buccal face, produced by a single movement (Fig 17A). In addition, one male cattle horn (#0033) has a chop mark at the base on its dorsal aspect. The mark runs perpendicular to the horn and is visible on the sheath, as the basal portion of the core is missing (Fig 17B). Two cattle upper molars present fire traces on part of the occlusal and lingual aspects of the tooth (#0082), and on part of the occlusal face (#0060). Whilst a horn sheath (#0034) presents a burnt area on its ventral aspect and inner surface of the sheath shaft, with slight fire related vitrification (Figs 16C and 18).

#### Coprolites

**Fig 19:**
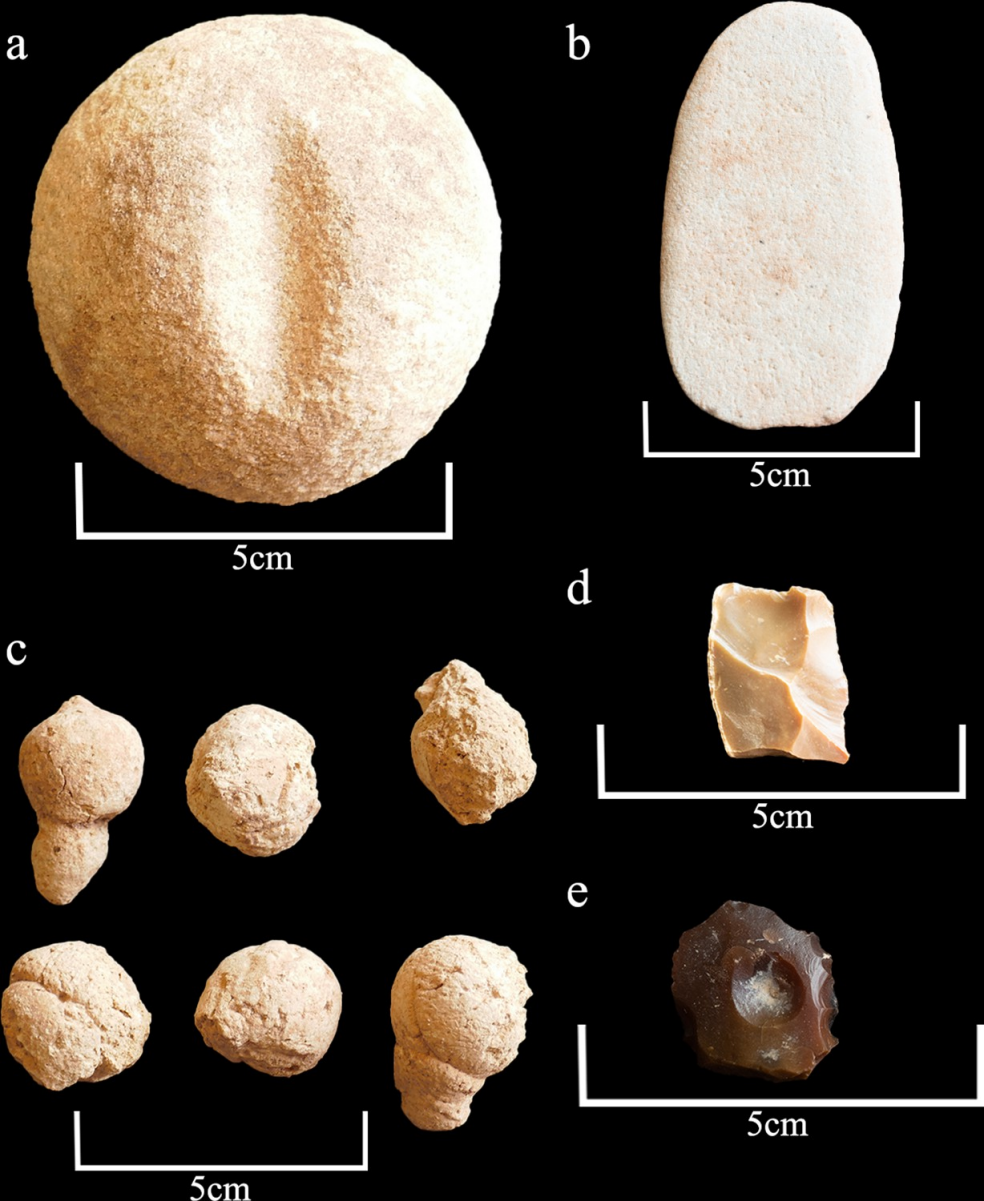
A selection of small finds from mustatil IDIHA-F-0011081. a: transverse grooved hammer stone (sandstone). b: polished grinder (sandstone). c: animal (hyena) coprolites. d: retouched flake. e: retouched side scraper.

Coprolites were found in Phase 1 (Fig 19C). The spherical shape of the pellets, with tip and dimple on the underside is comparable with the segmented faeces of hyaenids, formed by around 10 droppings each [38]. Their size around 15–25 mm suggests a possible attribution to the striped hyena (*Hyaena hyaena*; [39]), which was distributed in the area [13, 40]. Their pale colour, a consequence of feeding habits, which includes consuming bone, is consistent with this attribution and suggests an adult [39]. Moreover, hyenas typically defecate in latrines, with the small collection indicative of only a brief visit to the structure. Although hyenas are known to behave as bone collectors [39, 41], the skeletal assemblage within the chamber does not meet all of the diagnostic criteria for a hyena bone deposit [42, 43], and is therefore attributed to an anthropic agent. Indeed, compared to documented hyena assemblages the following differences are observed in the mustatil assemblage: the faunal spectrum (absence of bones of carnivores and hyena, small mammals, reptiles, birds, equids); the anatomical representation (absence of appendicular and axial bones); the absence of tooth marks and of digested bones.

### Artefacts from Mustatil IDIHA-F-0011081

In addition to the faunal remains, the main chamber and doorway yielded several anthropic artefacts, with the bulk of these finds recovered from Phase 1 (Fig 19). A planoconvex sandstone, transverse grooved hammer stone with a diameter of 6.6cm was found by the southern wall of the chamber in Phase 1. Both surfaces demonstrate evidence of abrasion; with this artefact finding good parallels in the Natufian and Neolithic horizons of the Levant, Mesopotamia and the Caucasus [44–49]. Examples from the Levant have been interpreted as multi-functional tools, as well as shaft straighteners for arrows [49–51]. A sandstone bifacial polishing stone was also identified in Phase 1. This artefact demonstrates evidence of use, with both faces revealing evidence of wear. In addition to the ground-stone artefacts, several lithics were recovered, including a small, backed tear-drop side scraper with re-touched edges (#0078) and two chert retouched micro flakes (#0080 and #0133); all of which were recovered from the main chamber (Phase 1), and the doorway (Phase 3A). These lithics presented Neolithic stone production characteristics well known from archaeological sites of Middle Holocene contexts [52–54].

### Radiocarbon dating

**Fig 20:**
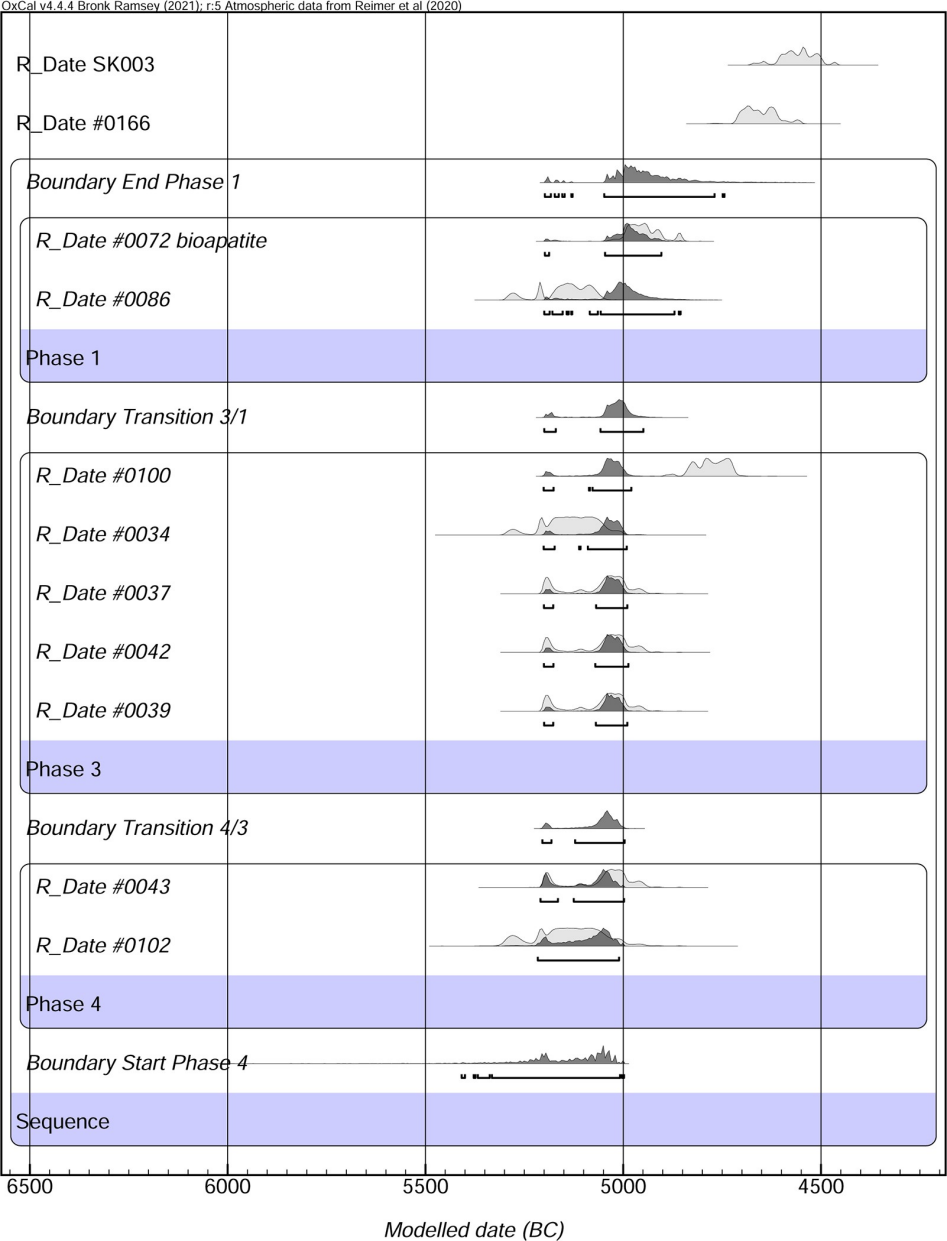
Radiocarbon dates for all features within site IDIHA-0008222 in cal BC. OxCal v4.4.4 Bronk Ramsey [21]; r:5 Atmospheric data from Reimer et al. [20].

A total of 11 samples were radiocarbon dated from site IDIHA-0008222, with nine obtained from the mustatil (IDIHA-F-0011081), and single examples from the cist (IDIHA-F-0011213) and hearth (IDIHA-F-0011091). All calibrated radiocarbon dates cluster around 5000 BC (Fig 20). The radiocarbon results for the mustatil (IDIHA-F-0011081) were modelled as three phases, as no samples were able to be dated from Phase 2. Slight later dates were obtained from the cist (IDIHA-F-0011213) and hearth (IDIHA-F-0011091) (Table 3). The dating results suggest a site occupation from 5307–5002 cal BC to 5056–4755 cal BC (Table 3). Individual calibration figures can be found in supplemental.

**Table 3:** Unmodelled and modelled (cal BC) radiocarbon data for all features within site IDIHA-0008222.

Lab ID	Sample ID	Context	Phase	F14C	±	^14^C age	±	Unmodelled (cal BC)			Modelled (cal BC)		
** **	** **		** **		** **	** **	** **	from	to	%	from	to	%
SANU-64603	#0102	Mustatil	Phase 4	0.4626	0.0031	6192	59	5306	4994	95.4	5217	5011	95.4
SANU-66609	#0043	Mustatil	Phase 4	0.4673	0.0013	6111	27	5208	5210	95.4	5210	4999	95.4
SANU-66606	#0039	Mustatil	Phase 3	0.4671	0.0013	6115	27	5209	4944	95.4	5201	4990	95.4
SANU-66607	#0042	Mustatil	Phase 3	0.4673	0.0014	6111	28	5208	4941	95.4	5201	4998	95.4
SANU-66605	#0037	Mustatil	Phase 3	0.4670	0.0013	6116	27	5209	4945	95.4	5201	4990	95.4
SANU-65412	#0034	Mustatil	Phase 3	0.4629	0.0021	6187	41	5292	5009	95.4	5202	4992	95.4
SANU-65411	#0100	Mustatil	Phase 3	0.4791	0.0014	5911	29	4846	4173	95.4	5202	4980	95.4
SANU-66604	#0086	Mustatil	Phase 1	0.4616	0.0013	6210	27	5296	5052	95.4	5200	4856	95.4
UGAMS-46488	#0072 (bioapatite)	Mustatil	Phase 1	0.471	0.0014	6050	20	5026	4848	95.4	5199	4904	95.4
UGAMS-51051	#0166	Hearth	N/A	48.6	0.13	5800	20	4718	4554	95.4			
UGAMS-46484	SK003	Cist	N/A	49.04	0.14	5720	20	4673	4461	95.4			

### IDIHA-F-0011081: The courtyard

**Fig 21:**
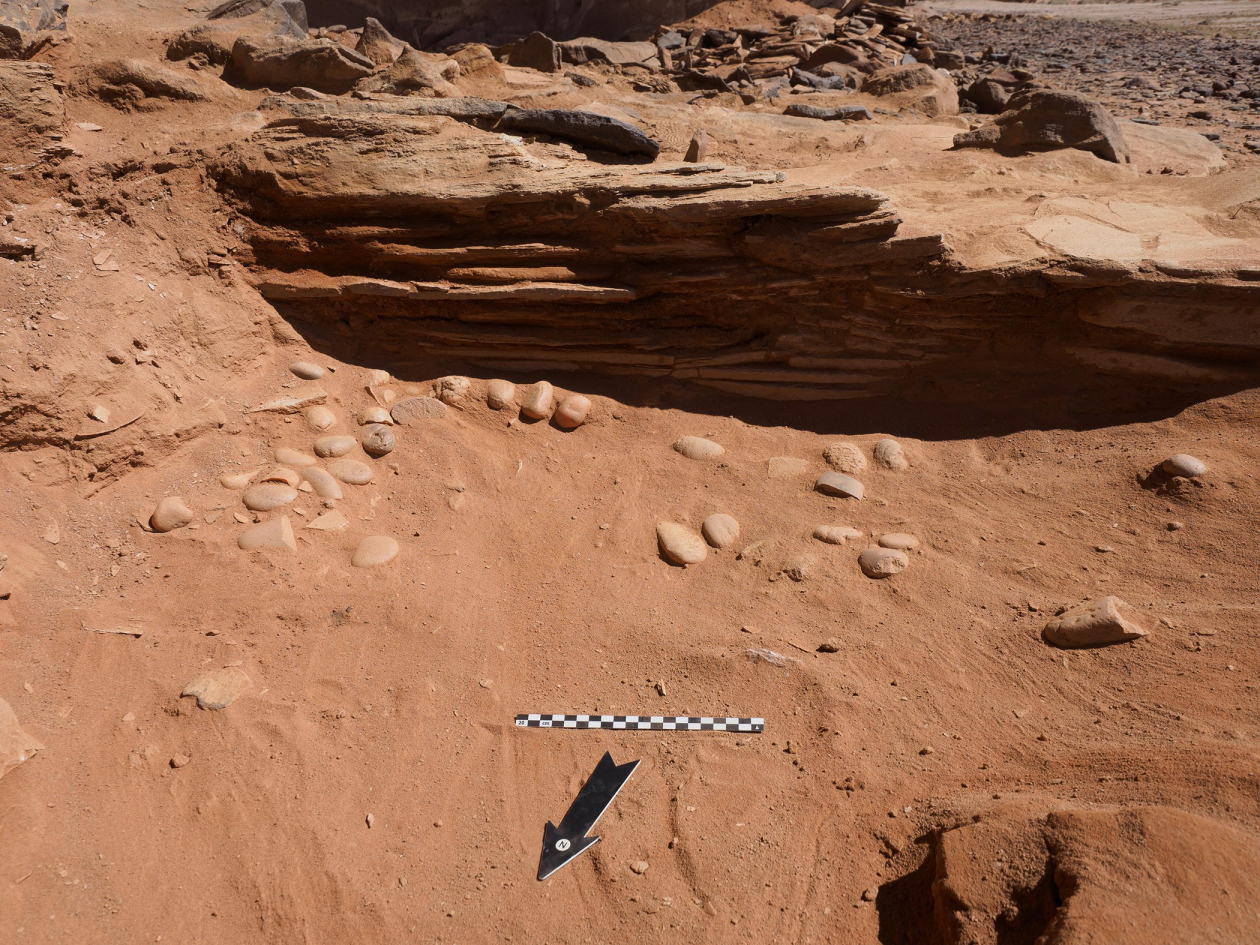
Riverstone surface associated with courtyard, further pebbles were set within the doorway.

A 2 x 2m sounding was executed against the western face of the head and the southern long-wall of the courtyard. Excavations in this area revealed a significant horizon of fill and rock collapse from the head. Immediately beneath this collapse were the remnants of a pebble surface (Fig 21). These river pebbles appear to have been set into the original ground surface level of the courtyard. Remnants of this surface were also identified in the doorway, suggesting this feature may have been more extensive than what is currently preserved. Beyond these areas of stones, a hard-packed surface had formed.

### IDIHA-F-0011213: The cist

**Fig 22:**
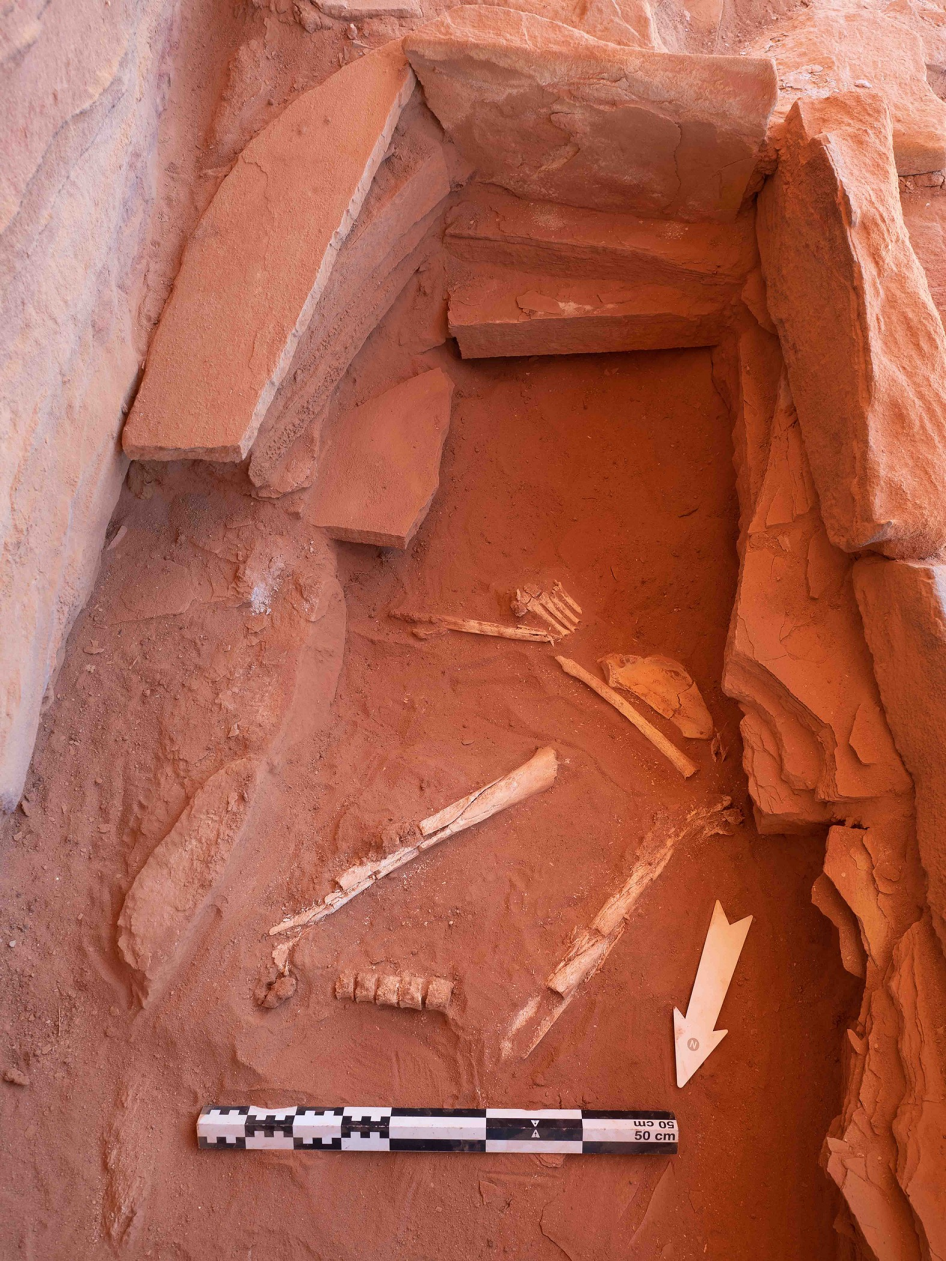
Partially articulated human remains located inside the cist IDIHA-F-0011213.

A small cist (IDIHA-F-0011213) was identified to the north of the head of mustatil IDIHA-F-0011081. Excavations revealed a sub-rectangular structure, with the external dimensions of 2.25 x 1.55 x 3.54 x 1.35m. Constructed from unworked slabs of sandstone desert pavement, this structure was sealed by wind-blown aeolian sand and sandstone debris from the rock overhang. Removal of this debris revealed three large in situ stone capstones in the southern end of the structure. In the north, these slabs were significantly degraded and had collapsed into the cist. Removal of the slabs revealed a deposit of orangey-red silty-sand, with the northern extent bioturbated. In the south, beneath the stone slabs were partly articulated and fragmented human skeletal elements (Fig 22). No other artefacts were found in association with these remains. Further clearance around the structure revealed that the southern end of the cist cut the northern end of the mustatil, suggesting that the cist post-dated the mustatil.

### Human remains from Cist IDIHA-F-0011213

**Fig 23:**
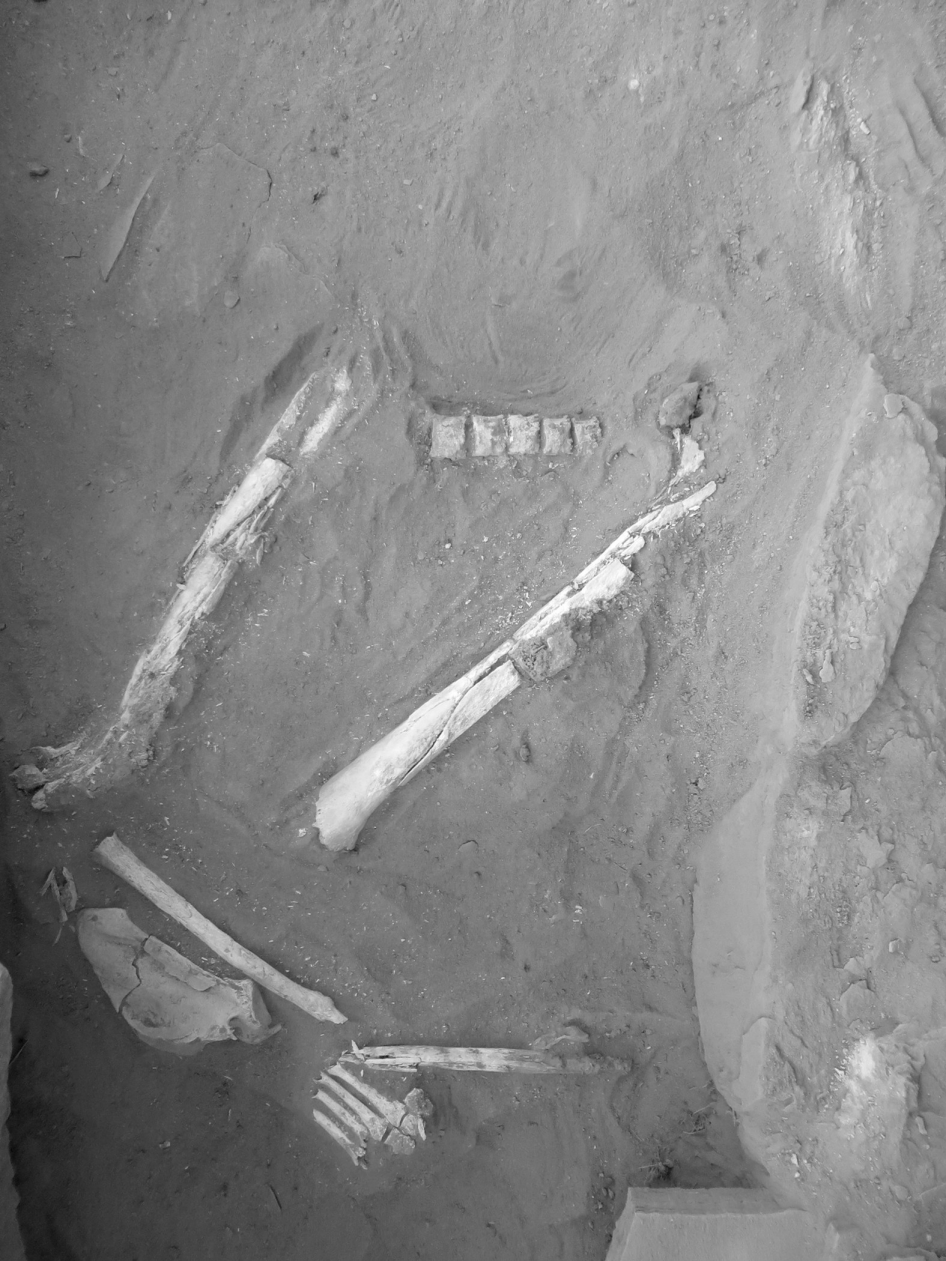
Detail of SK003, note the five thoracic vertebrae.

The primary objective of the anthropological analysis was to determine the minimum number of individuals within the structure and to undertake an anthropological assessment relative to demographics and life history (e.g., health status and potential cause of death). It was determined, based on no evidence of element duplication and similar demographic data, that the human remains recovered from the cist IDIHA-F-0011213 represent a single individual (SK003 –UGAMS-46484). Those remains present evidence of significant post-mortem disarticulation and fragmentation, and include tarsals and metatarsals of the left foot, five thoracic vertebral bodies (Fig 23), a left scapula, radial fragment, and two highly fragmented femora.

All of the bone recovered was considerably weathered and discoloured (reddish hue) from prolonged exposure to the dry silty sand associated with the depositional context. The bone is extremely friable and due caution was required to minimise further fragmentation during excavation, lifting, and in subsequent handling for anthropological assessment. It was evident that the burial had been displaced and disarticulated post-mortem; only two body parts were recovered in normal anatomical articulation: the first being a cluster of four metatarsals (Metatarsal two–Metatarsal five) and medial, intermediate and lateral cuneiforms of the left foot (Fig 22); and the second being the first metatarsal that was found inferior to the articulated foot bones with the first proximal phalanx. Fragments of the scapula and radius were discovered next to the articulated foot bones. Two non-articulated and incomplete/damaged femora (no distal ends) were excavated further north within the grave; those femora were not in anatomical position and are orientated in different directions, with the head of one pointing north, and the head of the other pointing south. A total of five articulated vertebral bodies were discovered to the north of the femora. Fragments of vertebral arches (laminae and processes) were found in close association to those bodies, albeit not articulated.

#### Osteobiography

**Fig 24:**
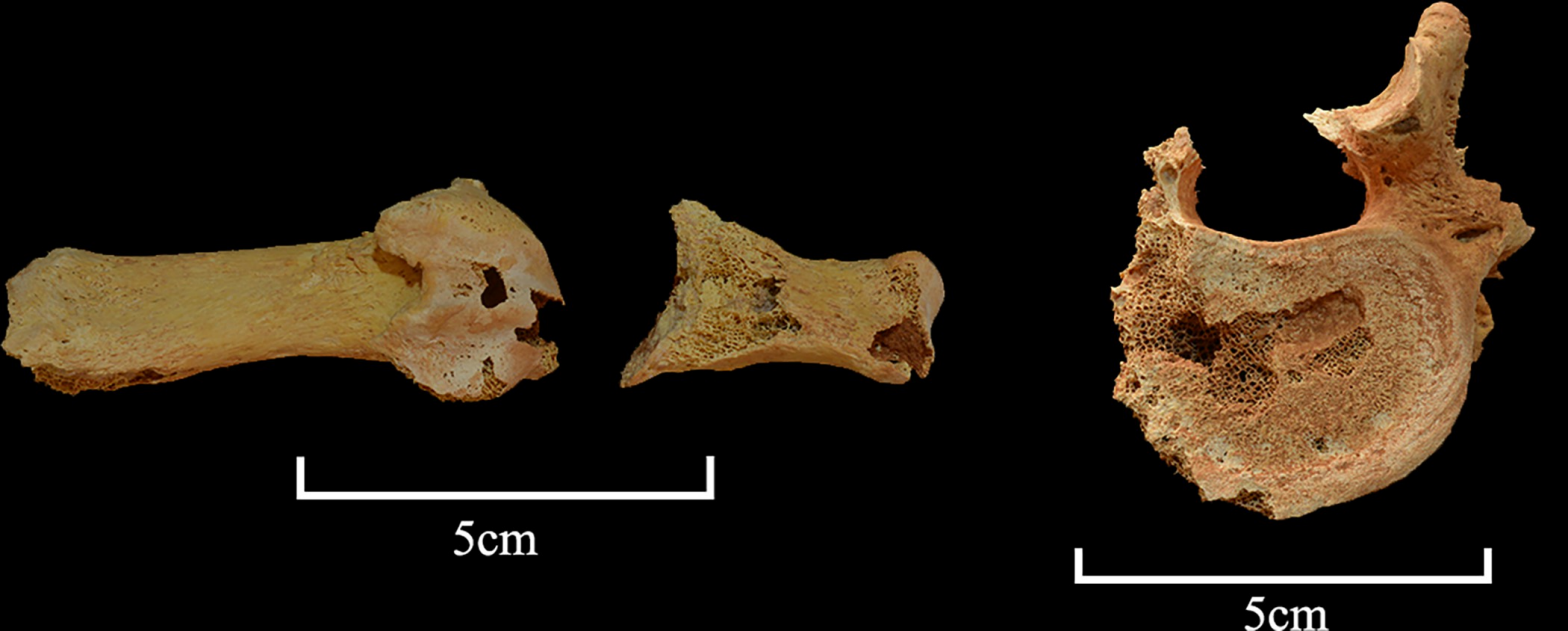
Detail of human bones marked by osteoarthritis.

A preliminary anthropological assessment was performed as part of the post-excavation analysis, although it is worth noting that this analysis is compromised by the highly fragmentary and incomplete nature of the individual. Male sex is estimated based on measurements of the scapula following Özer et al. [22], specifically the glenoid fossa. Adult status is assigned based on complete fusion of those secondary centres of ossification that are observable; e.g., femora and scapula [55]. It may be possible to infer that relative to the degenerative changes observed (see below) that this is not an overly young adult, instead tending toward a late-young to middle-aged adult (e.g., 30–40+ years of age), although such assertions are difficult to justify in the absence of larger comparative samples. Relative to the life history of SK003, there is macroscopic evidence of minor degenerative disease, with the anterior surface of three of the five vertebral bodies presenting evidence of early onset osteophytic lipping [56, 57]. Such metamorphoses are known from the published literature to be more common, but not exclusive to, middle-aged individuals [58, 59]. There is also evidence of Schmorl’s nodes, which are frequently cited as being associated with disc herniation [60] (Fig 24). The first metatarsal and articulating phalange present eburnation on the inferior surface of the metatarsal head, suggesting SK0003 likely suffered from osteoarthritis [61, 62] (Fig 24). No indications (such as perimortem skeletal trauma or systemic disease) pertinent to cause of death could be identified.

### IDIHA-F-0011091: Stone-lined hearth

**Fig 25:**
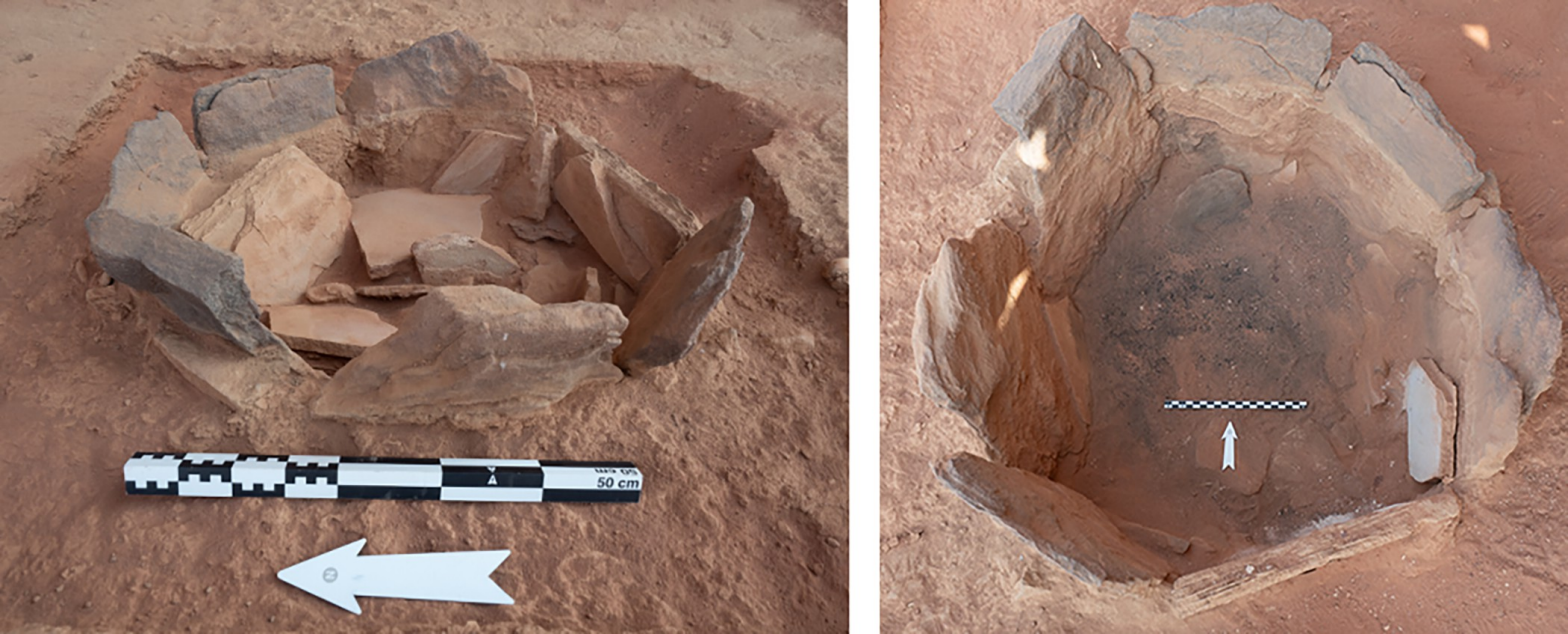
A Late Neolithic-early Chalcolithic hearth IDIHA-F-0011213.

In addition to the mustatil and cist, approximately 10m to the south of the mustatil was a small, stone-lined circular hearth (Fig 25). This feature had a diameter 66cm and was constructed from small sandstone fragments set vertically into the ground. Excavations revealed deposits of ash and charcoal, samples of which were sent for radiocarbon analysis (see below) and archaeobotanical analysis, which is currently ongoing. No other artefacts or detritus was found within this feature. Charcoal from this feature (#0166 –UGAMS-51051) was radiocarbon dated to 4717–4553 cal BC, several hundred years after the cessation of the mustatil (Fig 20; Table 3).

## Discussion

Collectively, the excavated material recovered from site IDIHA-0008222 allows for expansive discussions regarding the socio-cultural and economic aspects of the Neolithic period in the AlUla region. This assemblage is also of interest for ongoing preservation studies, which are critical to further understanding the taphonomy and environmental processes specific to this highly arid zone.

The faunal assemblage from mustatil IDIHA-F-0011081 represents a selection of anatomical elements pertaining to selected taxa, specifically horned taxa. As such, the faunal remains and their deposition can be considered as deposited portions, part of a ritualised act, with these remains amongst the earliest attestation of domestic cattle and goat in northern Arabia. In combination with the architectural, chronometric and osteobiographical evidence from site IDIHA-0008222, a number of observations regarding the nature and scope of cultic practices in the Neolithic can be outlined. These observations have further implications for understanding the development and role of ‘pilgrimage’ in the early mobile pastoralist groups of northern Arabia.

### The faunal remains: A summary

In terms of species representation, considering both NISP and MNI, the assemblage is predominantly composed of bovines (217 remains), in particular domestic cattle, followed by caprines (24 remains) including domestic goat, with gazelle accounting for a single element (Table 1). The 89 remains attributed to domestic cattle are amongst the earliest dated remains of this taxon in north-west Arabia; whilst the 128 specimens attributed to *Bos* sp. In all likelihood belong to domesticates. Some fragmentary *Bos* sp. Remains recovered at Jebel Oraf 2 have also been attributed to domesticates [63], rock art depictions also indicate the presence of cattle in the wider region [25, 64, 65].

Domestic cattle is also attested to in the steppic and arid areas of the southern Levant from as early as the 7^th^ millennium BCE, and in southern Arabia from the end of the 7^th^ to the early 6^th^ millennia BCE. The present assemblage would appear to be consistent with a dispersal into northern Arabia, from an adjacent region like the Levant [26, 66]. Cranial metrical data (Table 2) fits in the range of large specimens of Neolithic cattle from Kerma [67], and Shi’b Kheshiya [68]. Sex data suggests a high percentage of males, four out of the eight individuals, potentially targeted for their robust horns. Conversely, females may merely be underrepresented. Different age classes are also present, from sub-adults to older adults, notably absent are juveniles, individuals that were often selected for ritual slaughter and deposition [69]. However, dispersal and the fragility of juvenile bones may contribute to this absence in part, although two decidual teeth are preserved and are heavily worn. This mortality profile suggests the direct availability of livestock and a non-univocal choice of ages for deposition. Sub-adults provide a significant amount of meat and blood comparable to adults [70, 71]. Shape variability is consistent with domestic status, which may point to individual variation or to different animal populations. Moreover, combining the extant age and sex data, the high proportion of adult males is not indicative of economical (optimal) herd composition and culling practices [70–73], but points to a strong ritual component.

The domestic goat remains, are amongst the earliest examples of this taxon in northern Arabia. A further 19 remains can be attributed to undetermined caprines, with these most likely belonging to domesticates. Possible Neolithic goat remains have been identified around Jubbah [74]; with caprines believed to have been introduced to Arabia (possibly from the Levant) between 6800–6200 BCE [25, 52, 66, 75]. Age and sex determinations mirror those of the bovines. The available sex data indicates that males predominated, with two-to-three males, and no females identified. Age data also suggests that adults and sub-adults were selected, whereas juveniles, who are best consumed at this life-stage are not represented in the extant assemblage, unless preservation factors affected the sample [70]. Male survivorship into adulthood is not economically viable, with culling around 1.5 years generally undertaken to ensure herd security. This may explain the presence of the D4 tooth, belonging to an individual around that age [70–72]. Interestingly, this mortality profile contrasts with other Neolithic data-sets from southern Levant [76–80]. Despite the small sample set, the identification of three males out of the three MNI, is suggestive of ritual selection, and an emphasis on horned species and classes. The decidual tooth may also be a seasonal indicator of death. The birth season of ancient domestic caprines is believed to have occurred in the spring [81], as such the animal may have died between the spring and summer.

The female gazelle horn #0044 is the only attestation of wild taxon. Despite a predominance of males in the assemblage, #0044 indicates that females were also selected for ritual purposes. Gazelle are suited to arid and semi-arid environments and different species were historically distributed across the region [66, 76, 80, 82, 83]. Therefore, the incorporation of gazelle may be an indicator of climatic conditions with a tendency towards aridity. This is further suggested by the presence of the coprolites, most probably associated with the striped hyena, which lives predominantly in arid/semi-arid environments and in savannah areas, with their remains attested to the southeast on the Harrat Khaybar [40]. The low frequency of gazelle remains may indicate a mixed economy based on herding and hunting [25, 76, 77, 80, 84, 85]. Furthermore, research also indicates that hunting activity, particularly of gazelles was endowed with symbolic or socially prestigious values [cf. 86–92]. As such, this species may have been deliberately interred for ritual purposes.

#### Skeletal elements

The 247 faunal remains found in mustatil IDIHA-F-0011081 are only elements of the cranium. The absence of other skeletal elements suggests a strict selection, further enhanced by the absence of mandibles and mandibular teeth. Despite a possible dispersal, considering the significant number of individuals and the natural resistance of mandibles, these were not originally present in the assemblage. The absence of the post-cranial skeleton also appears to be intentional.

The chop mark at the base of the cattle horn, #0033, was aimed to separate the horn from the cranium. This practice is not related to food consumption but rather indicates the preparation of a specific element, the isolated horn. The remainder of the cranium may also have been deposited as a separate offering. At least six of the cattle and two of the goat sheaths were deposited as isolated elements. Moreover, the sheath is strongly attached to the horn core, and hard to remove when fresh, with extraction following specific procedures [93]. Therefore, isolated sheaths can be considered intentionally prepared portions, with a distinct meaning. In fact, they out number horns cores 20 to seven, indicating a specific function (Table 1). The partial goat skull, #N01, could be a horned frontal shaped by intentional procedure. However, a complete aigicranium is possible as caprine teeth and a goat petrous bone were found in the underlying context.

Teeth are also numerous (Table 1), in two cases (one cattle, one goat) they are part of maxillaries, with an additional cattle maxillary bone devoid of teeth also recovered. Teeth are better represented than horns, in the case of bovines (Table 1), due to their resistance. In two cases the maxilla bone was still attached, however, it cannot be excluded that maxillaries with teeth (the fragile bony part being less preserved) and isolated teeth were also deposited offerings.

Petrous bones are anatomically located in the caudal part of the cranial box, this suggests at least four cranial boxes were present in the assemblage (three bovines, also attested by a bovine basioccipital, and a single goat). The smaller quantity of cranial box fragments may be due to the fact that the walls of these elements are fragile, and as such are potentially less well preserved. When compared to the minimum numbers of different skeletal elements, petrous bones are not abundant (Table 1). Considering their resistance and compactness, it is possible that this discrepancy derives from dispersal/co-mingling, or could derive from a lower number of cranial boxes. Other portions of the cranium are also preserved, such as two frontal bones, a further fragmented goat left frontal with horn core base (located in the rear chamber), one temporal, three nasal bones and five incisor bones (with one cattle bilateral pair and one incisor attached to the maxillary). If the fragmentation is original, these parts could have been deposited as isolated elements. Nasal bones are fragile and are easily dislodged (see above), whilst incisor bones are thicker but are also attached to the skull through a suture, often lost with manipulations. One case of maxillary plus incisor is recorded. Isolated teeth could also easily have been dislodged from maxillaries. As such, combined, the remains may have originally been part of entire bucrania.

Based on the extant evidence, the deposited elements are:

1. the horn
2. the sheath
3. the horned frontal (possibly)
4. portions of the cranium (possibly)
5. the bucranium (possibly).

However, a specific focus on horns can be hypothesized. In quantitative terms, for each taxon, the minimum number of deposited elements is estimated. For cattle, the count is of six isolated sheaths, three horns, two maxillaries, three cranial boxes and 88 teeth. Matching the elements, the bucrania are at least four (based on incisor bone), with four additional right maxillaries. In terms of the caprines, the minimum number is two goat sheaths, one aigicranium, one goat horned frontal and one maxillary (that together would be one additional aigicranium). For the gazelle the count is one horn, likely deposited as single element.

#### Preservation

The preservation across the assemblage of coprolites and upper cranial elements was highly variable. This may be the result of environmental and/or anthropic actions prior to and during deposition of some elements. In particular, the preservation of 31 specimens of horn sheath is exceptional (Fig 16). Horn sheaths are the outer keratin shells of the horn cores, bone which outgrows on the cranium of ruminant artiodactyls. These sheaths grow progressively from the base, and are firmly attached to the core [29, 94]. The presence of cattle, goat and gazelle sheaths is extraordinary, as the quick degradation of keratin usually makes them invisible in the archaeological record [95, 96]. However, peculiar depositional conditions made possible the preservation of keratin in this instance. The most likely explanation for this is atmospheric dryness, leading to rapid desiccation, this process would also have produced the sheath fissuration and flaking observed on the specimens [94, 95]. Desiccation affected more directly the outer layers of the sheath leaving the core better preserved and closer to original appearance. The thickness of the tips also resulted in better preservation (11 preserved). In the case of mustatil IDIHA-F-0011081, substantial protection of the deposit came from the rock overhang, which potentially created a microclimate, acting as shield from atmospheric events, in particular from rainwater that facilitates degradation. The overhang may also have created a more stable temperature, with seasonality of the deposits also potentially playing a role. Indeed, studies have demonstrated that the heavy degradation of horns occurs in the first 3–12 months after burial [97]. However, the potential for other unknown anthropic modes of preservation cannot be excluded.

Despite good preservation, deformation was also present. Two sheaths are deformed: #0032 has its basal portion broken and compressed, as well as a transversal concavity, damage, or wear at 200 mm from the tip, as on #0043 (280mm from the tip). The deformation of the *Bos* sp. sheath #0032 may be related to the plastic properties of keratin, which can be deformed by heat and made fragile by water [93, 95], or anthropic agents. The transversal wear of #0032 and #0043, may be due to post-depositional factors on sheath annulations [29] or to pre-depositional damage (produced during the animal’s life or related to some way of storing the sheaths before deposition). In instances where a difference in preservation was observed within an individual specimen, exposure of the top zone to post-depositional agents is likely a factor [98]. Holes were observed on six specimens (bovine and caprine), attributed by S. Vanin (personal communication) to the action of invertebrates, this is currently undergoing a focused study to clarify diagenesis.

Preservation of the coprolites is also consistent with desiccation. Their outer layer solidifies rapidly, keeping the original shape. Coprolite preservation depends on several factors (faeces composition, temperature, humidity, biotic agents and soil), but carnivore coprolites are better preserved due to the higher mineral content (bone) of their diet [39].

Compared to the horns, bone preservation can be considered poor. Phases 1, 2 and 3A were characterised by the poorest preservation with very brittle bony elements in weathering stages 3 to 5 [99]. In some cases, both bone and sheath disintegrated when excavated: altogether 38.5% of bone remains in these phases had lost all structural integrity and disintegrated during the course of removal, with variable preservation in Phase 3B (1.8%). This may indicate that water was able to pool within the main chamber early in the sequence, but this would hamper the preservation of sheath (e.g. #N01 had both bone and sheaths present but had lost all structural integrity). As for coprolites, hyena scats are known to maintain their resistance and compactness even after soaking for weeks in water without previous desiccation [100]. This change in the conditions within the overhang may be indicative of increased aridity during the life of the mustatil.

In summary, further taphonomic studies of the assemblage are necessary, as well as investigations on the possible treatment of the sheaths prior to deposition. Chemical analyses of the soil will also be required to understand this fundamental factor in the harder tissue diagenesis, as this may have contributed to the brittle conditions of the faunal remains in the earlier phases. Understanding the changing environmental conditions and the methods and means of preservation used at site IDIHA-0008222 will further inform our understanding of the cult practice enacted at the site.

### Cult and ‘Pilgrimage’ in North-west Arabia during the Late Neolithic

The monumentality of mustatil IDIHA-F-0011081 and the associated deposition of animal horns and upper cranial elements, along with the inclusion of fire, in close association with a standing stone, suggests that the structure had a supra-domestic or cultic function [86, 101, 102]. Although, the nature and meaning of the rituals associated with these remains are unknown, it appears to have been focused around the large central up-right stone (A), located in the east (rear) of the main chamber, with almost all of the faunal elements clustered around this feature. Based on the size, positioning and stone choice we have interpreted this up-right stone (as well as B and C) as a betyl (“house of the god”). As such, although there may be other interpretations, we hypothesise that the standing stones (betyls) from mustatil IDIHA-F-0011081, particularly stone A, may have functioned as a mediator between humankind and the divine, acting as a proxy or a manifestation of an unknown Neolithic deity/deities or religious idea, to which the faunal elements were deposited as votive offerings. However, it should be noted that based on our extensive ground survey evidence, of more than 80 mustatils, we do not believe that betyls featured in all examples of this tradition. Despite this caution, the presence of at least three betyls is highly significant, with these examples amongst the earliest chronometrically dated (late 6^th^ millennium BCE) instances of a tradition, which was to become a hallmark of the pre-Islamic ritual landscape of Arabia [103]. Indeed, the religion of pre-Islamic Arabia has been described as a “cult of the betyls”, with up-right stones, natural rock outcrops and other portable idols revered as a manifestation of, or as a house of the god(s) [103]. These stones were also believed to have been associated with animal sacrifices and blood offerings, which were poured over the betyl, in order to garner the favour of the god(s), and to ensure fecundity [103]. Ritualised hunting and feasting, of both wild and domestic taxa also played an important role in this tradition [101], as it did elsewhere across the Neolithic [cf. 86–92]. Although these practices have generally been ascribed to southern Arabia and a later time period [103], on the basis of the radiocarbon evidence from mustatil IDIHA-F-0011081 it is possible that such beliefs have their origins earlier than previously supposed.

The apparent ritual dedication of both wild and domestic taxa, along with the predominance of cattle, based on the identifiable data, in mustatil IDIHA-F-0011081 is of note. Indeed, over the last few decades scholars have highlighted the ritual importance of cattle (particularly bucrania) and cattle sacrifice in the Early-to-Middle Holocene cultures of the Middle East, Africa and Europe [68, 103–115]; and to a lesser extent goats and gazelle [87, 88, 104]. Currently, mustatil IDIHA-F-0011081 offers some of the earliest extant evidence for the existence of a cult which incorporates the offering/dedication of both wild and domesticated taxa in Arabia; predating the earliest known (published) example by almost 900 years [112]. Excavations at Shi’b Kheshiya in the Wadi Sana, Yemen, revealed an oval ring of 42 cattle skulls positioned nose-down and inward facing, orientated around a central skull [112]. To the west of the skull ring were two up-right stones, although these predate the construction of the skull ring, they echo the betyls of mustatil IDIHA-F-0011081. Radiocarbon assays indicate a *terminus post quem* of 4457–4263 cal BC for the skull ring and 5308–4736 cal BC for the upright stones or betyls. Interestingly, the betyls from Shi’b Kheshiya appear to be roughly contemporary with the betyls from mustatil IDIHA-F-0011081 [116], perhaps suggesting a wider Peninsula phenomenon. McCorriston *et al*. [112, 115] have interpreted the ring as a marker of ritualised feasting and a monument to increased territoriality, specifically the control of pastoral resources. We have previously suggested that the mustatil tradition functioned in a similar manner [6]. This is implied by the monumentality of these structures and the fact that they were constructed to a preformulated and consistent plan, with comparatively little architectural differentiation discernible across the geographic distribution of this tradition, at present more than 300,000km^2^ of northern Arabia [6]. Indeed, roughly contemporary monumental structures, interpreted as territorial markers, have been identified in northern Arabia at Dûmat al-Jandal, which was also found in association with maxillary bovine teeth and limb bones [54]; with slightly earlier (and smaller) examples identified in the Dhofar region of Oman [117]. A similar hypothesis has also been proposed for the monumental cattle burials of the Late Neolithic Sahara [109].

Although temporally and geographically dispersed, these varying monument types were intended to visibly mark the landscape, albeit as culturally and regionally distinct manifestations of territoriality. However, the widespread distribution and typological uniformity of the mustatil tradition indicates that the symbolism and perhaps meaning of these structures was potentially understood across a vast geographic area. This contrasts with earlier assertions that the Neolithic nomadic populations of north-west Arabia left little material evidence of contacts between groups [52, 75]. Instead, it implies that northern Arabia to the Rub’ al-Khali and beyond was characterised by one of the world’s earliest and largest ritual landscapes, potentially linked by a common belief and ritual understanding. This assertion is supported by the identification of roughly contemporary deposits of upper *Bos* sp. molars (consistent with mustatil IDIHA-F-0011081) and 12 remains of the appendicular skeleton of three oryx, in a partially looted mustatil (NEF-8), located more than 100 km to the north-east of mustatil IDIHA-F-0011081, on the southern edge of the Nefud Desert [5]; although it should be noted oryx and limbs are absent from all excavated mustatil in the AlUla region.

Whilst it is not possible to conclusively ascertain when and where the sheaths, horns, and horned frontal were prepared, the preserved elements of the skull provide notable evidence for their treatment. The presence of fragile parts, such as the nasal and incisor bone, suggests several possible explanations. One is that the skulls were deposited fresh, and as such were prepared after culling in the context of the ceremony itself, either at the mustatil or in close proximity. This would suggest that the ritual slaughter of animals and the preparation of bucrania was a fundamental aspect of the ritual. Based on this hypothesis, at least four to eight cattle and two goats were slaughtered in an undefined number of events, their skulls deprived of the mandibles and skinned on site. As mentioned previously, the caprine D4 would place the death of this animal between the spring and summer, such deposition is consistent with the high-level of sheath preservation. A further explanation is that soft tissue was still present (or dried) on the skull, to keep the cranial elements in anatomical connection. In this scenario, the preparation of the skulls may have occurred in an undefined moment prior to deposition, but possibly not long before. Although we are unable to determine whether the deaths of these animals were episodic, natural or deliberate, the deposition of these elements, regardless of when and where they died, appears to be key to the events at mustatil IDIHA-F-0011081. Moreover, the preparation of offerings beforehand gives a deeper meaning to their preservation and transportation, and expands the significance of the ritual practice beyond the timeframe of the deposition ceremony itself.

Due to the number and age of the animals slaughtered and the presence of fragile cranial elements, suggestive of fresh skulls, and anthropic marks indicating specific processing practices, we hypothesise that ritual feasting also played a role at mustatil IDIHA-F-0011081. Although the episodic killing/natural death of these animals cannot be excluded, we believe that that this hypothesis is the most likely scenario for the faunal deposits. Regarding the observed anthropic traces, the cut marks on the molar #0056 are indicative of cheek skinning [91], perhaps for a facial hide [e.g. 70, 84]. Despite the absence of other anatomical elements, it can be hypothesised that these animals were skinned and consumed. Furthermore, if the skull fragmentation is original, this may be indicative of intentional breakage, potentially associated with brain consumption. Fire-related traces were also present on two molar teeth, although not directly related to meat consumption, suggesting a possible role for fire. The localised spread of these traces indicates only a short contact with fire. This also applies to sheath #0034, which exhibits localised burning, and is not calcinated nor cracked. The glass-like substance observed is, an effect typically produced by fire, generally occurring within a few minutes of contact [118]. Contact with fire is also evident within the sheath. The dark grey/black sheaths found in Phases 3A and 3B also appear to have come in contact with ash or fire, although direct and prolonged exposure to fire appears to be unlikely [118].

The slaughter of at least four to eight cattle and two goats would have resulted in a considerable amount of meat. Analysis of the cattle remains from Shi’b Kheshiya suggests as many as 5000 individuals could have been fed from the slaughtered cattle [25, 111]. Likewise, evidence from the roughly contemporaneous cattle ‘burials’ of the Sahara indicates that hundreds of participants may have been involved in the consumption of these animals [109]. Whilst, at the earlier Levantine PPNB site of Kfar HaHoresh, between 500-2000kg of meat was produced, potentially feeding up to 2500 participants [110]. Although comparisons to the above are of note, nowhere near as much meat would have been generated at mustatil IDIHA-F-0011081, as the ritual activity at this site appears to have occurred over a period of time (albeit short), rather than as a single event. Despite this, the slaughter of a single sub-adult bovine would have resulted in approximately 100-120kg of meat and 7l of blood [70]. Based on the work of Goring-Morris & Horwitz [110] and taking into consideration the upper range of 120kg for a sub-adult bovine, the slaughter of a single bovine would have been enough to feed approximately 600 individuals, based on an estimate of 200g per person. Even if larger portions were consumed, or if the animal was feasted on over multiple days, it suggests more than 100 individuals could have been fed in this ritual activity.

If ritualised feasting was a component of the mustatil tradition, notably absent is the detritus associated with feasting, such as pits and waste, with only horns and bucrania recovered from the chamber. No faunal evidence has been identified in any of the excavated areas in the courtyard; although this does not preclude the possibility that such detritus is present in un-investigated areas or was not preserved (as may happen to faunal remains left on the surface [119]). Moreover, the possibility remains that such debris was removed, with the site cleaned after use. The possibility that the courtyards of the mustatil were ritually cleaned is potentially supported by survey evidence. The AAKSA project has ground surveyed more than 80 mustatils, with limited surface artefacts identified in only 10 examples.

Feasting in prehistoric societies was highly ritualised and a key component in constructing and consolidating kinship ties and communal social identities [103, 112, 120–122]. These events involved considerable preparation, as well as surplus accumulation [123, 124]. Indeed, as Hayden [123] asserts feasting is concerned primarily with the production, control and distribution of surpluses, and public displays of largess. As such, the etic rationale for feasting can include the mobilisation of labour, fostering of social cohesion, the forging of alliances, risk reduction, and the creation and maintenance of social and/or political power [123, 125]. All of these facets are visible in the mustatil tradition, not just in the possible slaughter of more than one animal, a sizeable proportion of community wealth; but also, in the construction of the mustatil itself. Indeed, the size and sheer amount of stone and labour involved in the construction of these features indicates that multiple groups of people were involved in the construction of the structures. As such, this potential communal building programme, with the deposition of selected offerings and possible ritual slaughter and feasting may have been undertaken as a means of developing and maintaining social cohesion amongst the differing nomadic pastoral communities of the region, as well as defining territories and access to pastoral resources [103, 112, 122]. It also suggests a highly complex and structured social system, in which animals held both an important symbolic and subsistence role.

Despite the amount of meat generated by the sacrifice and the number of individuals it would have taken to construct the mustatil, the main offering chamber is comparatively small, measuring 3.10 x 2.80 x 2.5 x 2.85m in size. Due to this small size, the chamber can accommodate less than five individuals comfortably. This leads to the question as to how many individuals were involved in the deposition of the faunal elements. Although the chamber could only have held a limited number of individuals, the courtyard, to the west, could have accommodated a much larger gathering. Indeed, the chamber is visible across the eastern end of the courtyard.

### Early ‘pilgrimage’ and memorialisation in the Arabian Peninsula

Like McCorriston [103], we define pilgrimage as “a journey to a sacred space to participate in a system of sacred beliefs”. In addition to the physical act, pilgrimage, like feasting, can also be used to affirm collective identity and reinforce specific social roles [103], facets we believe are evident at site IDIHA-0008222 and within the broader mustatil tradition. As such, the identification of at least three-to-four stratigraphically distinct offering phases or events represents one of the earliest examples of ‘pilgrimage’ or shrine revisiting currently identified in the Arabian Peninsula. Although based on the radiocarbon data, these events appear to have occurred over a relatively short period of time, perhaps only a generation or two. This assertion is supported by our further four, unpublished mustatil data-sets. As mentioned previously, the sheer amount of labour and community focus/investment involved in constructing the mustatil IDIHA-F-0011081, and many of the mustatils of northern Arabia, suggests that these structures held a key role in the lives of these early herding communities. As such, the revisiting and pilgrimage to these structures, like feasting, may have been crucial to maintaining socio-cultural and economic ties between families and wider community groups, brought together by this ritual activity. Similarly, the episodic use of mustatil IDIHA-F-0011081 and indeed the wider distribution of the mustatil tradition argues for a vast and deeply rooted *habitus* [cf. 126–128]. This *habitus* and the possibility that the mustatil were only used over a generation or two may go some way towards explaining the sheer number of mustatils, more than 1600 in number, and their vast distribution across more than 300,000km^2^ of northern Arabia. Perhaps these structures were only permitted to be used for a specific period, before being de-commissioned, with each generation or subsequent generation required to construct a new mustatil. However, the sheer number of examples suggests a sizeable population inhabited northern Arabia during the Neolithic.

The location of mustatil IDIHA-F-0011081 deep within the dense sandstone canyons suggests the feature was not intended to be highly visible, a facet also possibly suggestive of pilgrimage. As mentioned previously, this structure is located 4km from the nearest, visible (undated) settlement. Although it should be noted that other more ephemeral types of occupation, such as hearth sites, located closer to the mustatil, cannot be excluded. The absence of occupation in the immediate vicinity of the structure is of note, it suggests that individuals travelled to the site and that its location was chosen for a specific reason. Based on remote sensing and low-level aerial reconnaissance, most of the mustatils across AlUla County were not constructed near areas of habitation, or if they were, these settlements could not have accommodated a population large enough to build the structure. In contrast, many of the mustatils on the Harrat Khaybar are located in more readily visible locations, often close to areas of habitation, such as large stone mounds, camps, and enclosures, although most of these features remain undated. Despite this, the Harrat Khaybar appears to have been densely occupied during the Neolithic, with more than 400 mustatils and more than 900 kites identified across this vast basalt landscape [4, 6, 129].

Moreover, as mustatil IDIHA-F-0011081 was essentially hidden in the sandstone canyons it indicates that the knowledge of its location was maintained over time. This is supported by the fact that the structure appears to have maintained a degree of significance after its decommissioning. This is suggested by the construction IDIHA-F-0011213 (cist), and to a lesser degree IDIHA-F-0011091 (hearth). The construction of IDIHA-F-0011213 and the interment of individual SK003, some 400–500 years after the final phase, suggests that a strong tradition of social memory and memorialisation was maintained across the Neolithic and Chalcolithic periods. Social memory and memorialisation appear to have been a key component of these periods in the AlUla region, with this taking the form of monumental collective burials, some of which span a 600-year period [130]. This later emphasis on collective burials, which were also used as territorial markers, broadly corresponds to the end of the mustatil phenomenon; with this transition potentially marking a change in the way territoriality was expressed from ritual to funerary during the mid 5^th^ millennium BCE.

Finally, the age (30–40+) of the individual interred in IDIHA-F-0011213 (cist), and the fact that partial, semi-articulated remains were deposited, may suggest that the individual was of some importance. Whilst, the nature of the burial indicates that the deceased died elsewhere; with their remains transported to the site, when soft tissue was still in place. This transportation, in the absence of visible nearby occupation, suggests this was a deliberate act, with this site chosen for a specific reason. One possibility is that this individual was interred here as a means of maintaining a territorial link with the past. In subsequent periods, the reuse of earlier tombs and structures were used as a means of co-opting territorial legitimacy and negotiating identity [cf. 130–132]. It is possible that this burial functioned in a similar manner, with the individual interred in cist IDIHA-F-0011213 as a means of maintaining a link with the ancestral past, the earlier mustatil tradition and all it entailed.

### Cult, herding, environment and landscape in North-West Arabia during the Middle Holocene

The faunal remains from the mustatil IDIHA-F-0011081 testify to the diffusion of both cattle and goat herding in north-western Arabia in the late 6th millennium BCE. It is also suggestive of mixed herding practices, supplemented by hunting and gathering [cf. 76, 84]. Contemporary mixed herds are attested to in the arid and semi-arid regions of the southern Levant, and southern Arabia, where a reliance on cattle herding appears to have been of greater importance [see 23–25, 76, 77, 80, 84, 87, 133, 134]. The presence of herding, in particular of cattle, is also indicative of more humid environmental conditions. It suggests the region carried sufficient pasturage and water resources for cattle herding, the latter of which is crucial due to cattle’s requirement to be watered every two-to-three days [25, 49]. At the same time, the inclusion of gazelle, usually found in arid and semi-arid landscapes, points to a general tendency towards aridity, all of which is consistent with the Holocene Humid Period [52, 135–140]. The Holocene Humid Period traditionally dated to ca. 8600–4000 BCE is believed to have been caused by the northward movement of the Inter-Tropical Convergence Zone and the Indian Ocean Monsoon, as well as the potential eastward expansion of the East African Summer Monsoon. All of which resulted in increased summer rainfall, significant surface runoff and a charging of the regions aquifers [141–147]. Northern Arabia is also believed to have been impacted by the winter rainfall patterns of the North Atlantic and Mediterranean climatic systems [148, 149]. Yet, despite increased rainfall, episodes of climatic variation and aridity were known to have occurred. Until recently very few palaeo-climatic records were available for northern Arabia [see 65, 135, 138, 139]. These reconstructions indicate that there were local variations in groundwater levels and rainfall, with drought episodes and a return to shrublands. Indeed, recent research at the Tayma palaeolake has suggested that the Holocene Humid Period was much shorter in this part of the Arabian Peninsula, dating to ca. 6800–5900 BCE, with the phase gradually ending by 5800 BCE [150]. This research indicated that the period between 5800–4800 BCE, which correlates with extant radiocarbon evidence for the mustatil tradition, was marked by an increasing aridity and aeolian influx [150]. Whilst broadly corresponding to conclusions made regarding the preservation of organic material within mustatil IDIHA-F-0011081, this data appears to be somewhat incongruous with the significant bovine deposits recovered, animals that need ready access to secure water sources. Likewise, evidence from the Jubbah Oasis suggests that there was episodic lake formation from as early as 6500 BC, with a high stand around 5300 BCE [151, 152]. Moreover, the large radiocarbon dataset amassed by the AAKSA project, suggests that the period between 5800–4000 BCE was marked by an explosion of settlement across all major environmental zones of AlUla County. Although no published palaeo-climatic data is available from AlUla, the possibility remains that northern Arabia was marked by a series of micro-climates or fluctuating patterns of rainfall that made both mobility essential and herding viable. Likewise, if this period (5800–4800 BCE) was indeed marked by increasing aridity it is possible that the mustatil tradition arose in response to these changing climatic and environmental realities. As such the rituals undertaken in the mustatil may have been associated with life-cycle events, particularly fertility and the seasons. This hypothesis is potentially supported by the fact that at least one animal appears to have been culled between the Spring and Summer [cf. 101, 122]. Similarly, cattle, particularly bulls have also been associated throughout antiquity with fertility, rain and masculinity/warrior tradition [118], such as the Bronze and Iron Age deities, El, Ba‘al, and Hadad [153]. As such the emphasis on sacrificing/offering cattle at IDIHA-F-0011081 may in this instance have been undertaken as a means of ensuring the continuation of the rains and securing the fertility and security of the Late Neolithic communities of northern Arabia. The mustatil may therefore have fulfilled both a sacred and profane (social) function.

**Fig 26:**
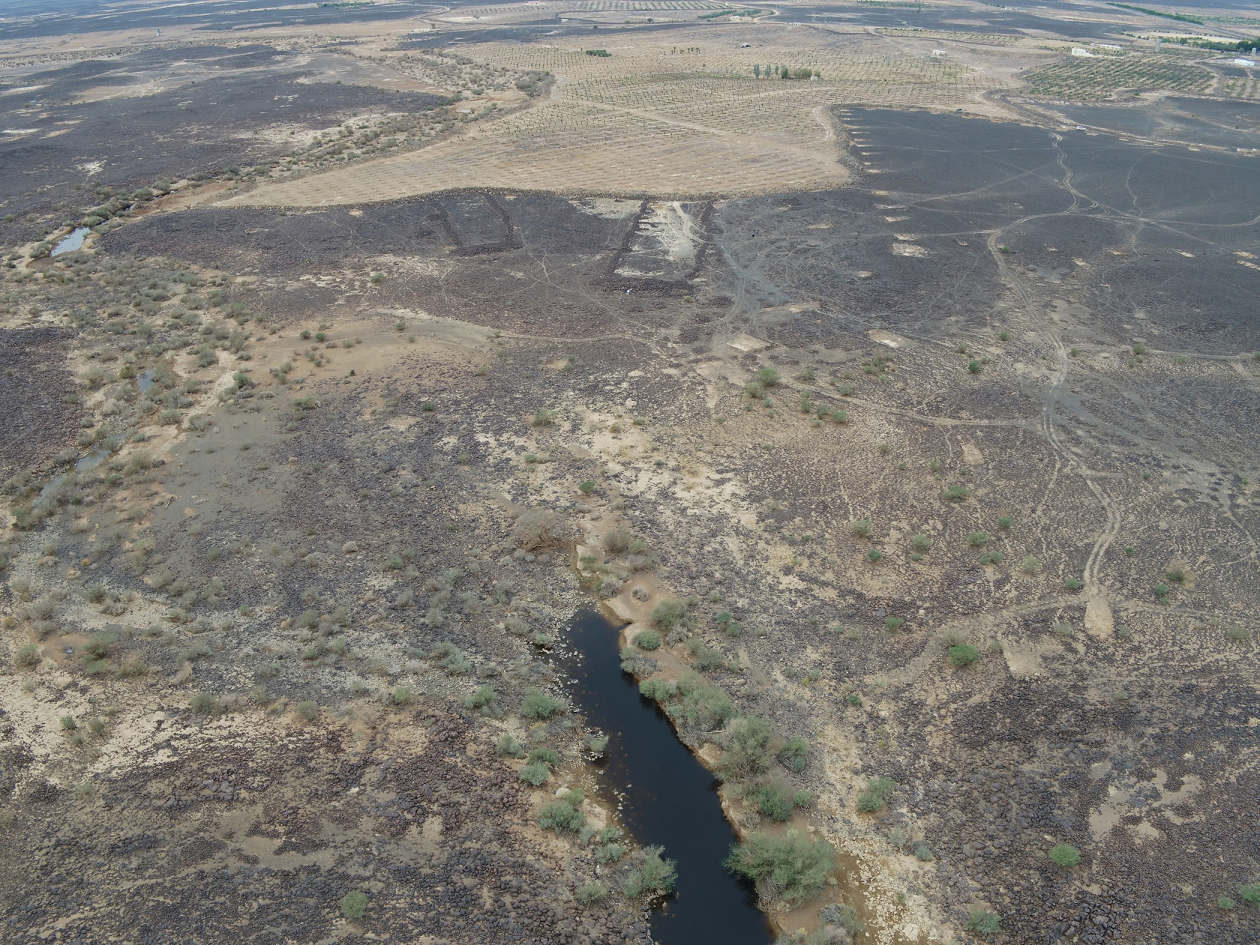
Two mustatil at site IDIHA-0030862 in Khaybar County orientated (base) towards a body of standing water, photo orientated west.

**Fig 27:**
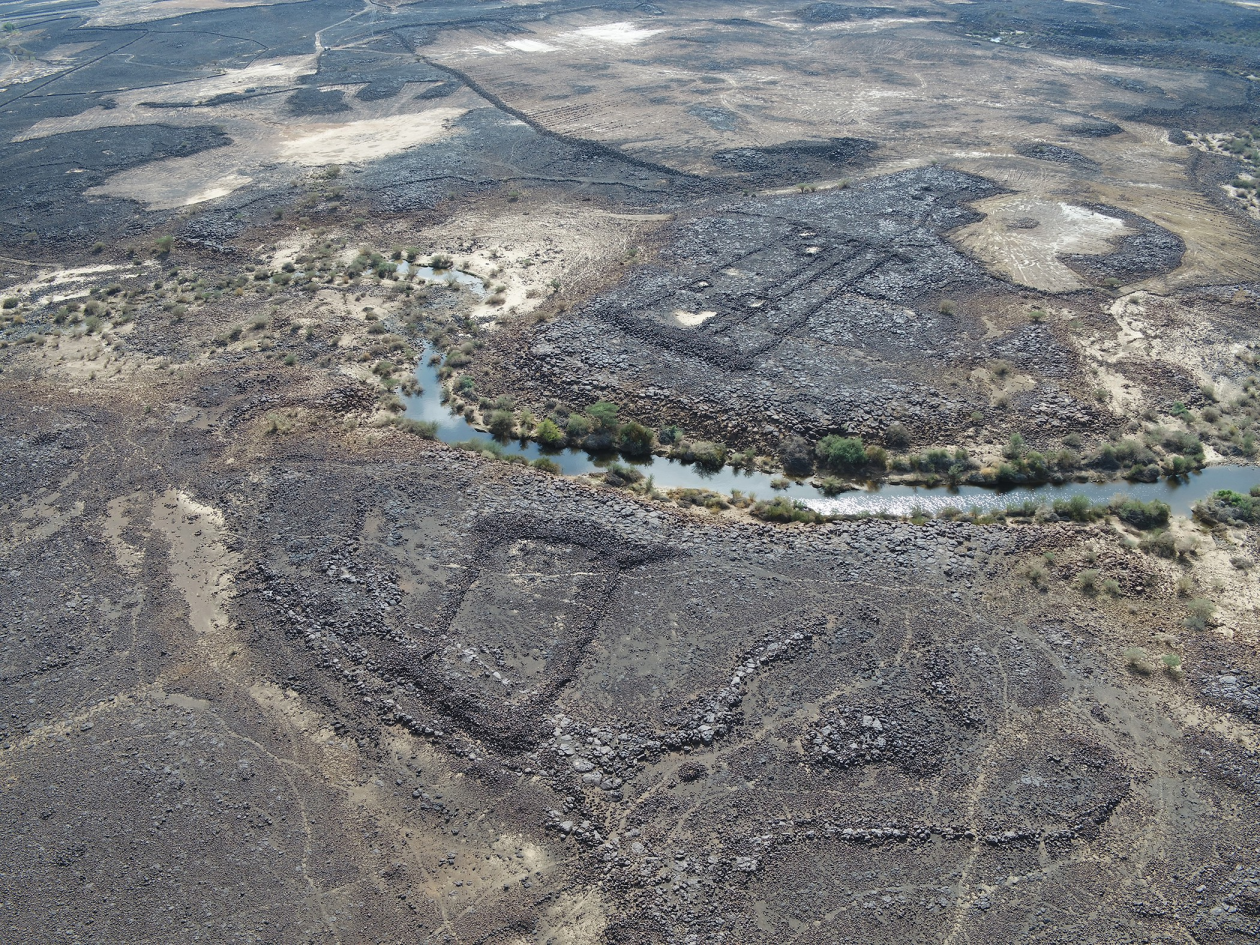
Three mustatil at site IDIHA-0030914, orientated (base) towards a small seasonal wadi in Khaybar County, photo orientated south-west.

**Fig 28:**
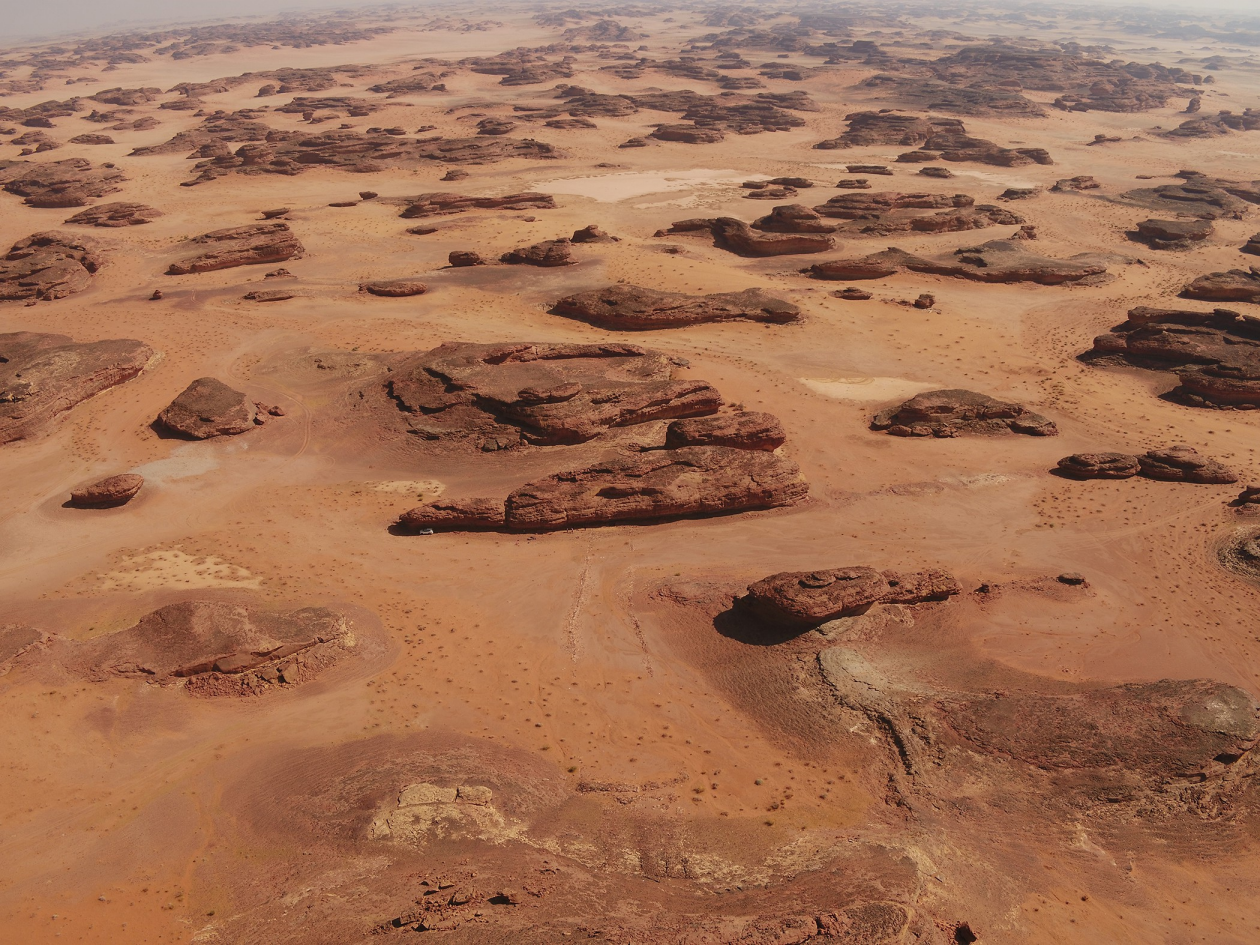
Mustatil IDIHA-F-0011081 orientated (head) towards a playa located to the east, photo orientated east.

The potential association between mustatils and rain/water is supported by our recent aerial survey of numerous mustatils after heavy rain on the Harrat Khaybar and our on-going multi-scalar GIS study of this feature type. This work has revealed that the majority of these structures were orientated towards or were constructed in close association to bodies of water, such as wadis, playa or ‘qa (Figs 26 and 27). Likewise, mustatil IDIHA-F-0011081 appears to be orientated towards a playa, 700m due east (Fig 28). As such, it can be hypothesised that if specific species were slaughtered as a means of ensuring fertility and potentially the continuation of the rains, it is possible that the positioning of the mustatil themselves was of equal importance, with these structures located near areas of key pasturage and water. Although articulating the precise relationship between mustatil and water sources is currently the subject of a larger multi-scalar GIS study by our project, it offers an intriguing and key avenue of future research.

## Conclusions

The excavations at site IDIHA-0008222 have revealed new insights into the economic and ritual landscapes of the Late Neolithic in north-west Arabia in during the 6^th^ and 5^th^ millennia BCE. The offering of both wild and domestic taxa indicates that both hunting and herding were conducted in AlUla throughout this period. The presence of multiple species of domestic taxa, may also indicate that mixed herding was practiced. Furthermore, the predominance of cattle, within the identifiable corpus, suggests that the surrounding region was characterised by sufficient vegetation and accessible water to sustain cattle herding, with this potentially suggesting the continuation of the Holocene Humid Period in this part of north-west Arabia, contrary to more recent analyses from Tayma [cf. 150].

With the exceptional state of preservation, the remains found within the mustatil of IDIHA-F-0011081 provide preliminary evidence for the ritual activities practiced within these monuments, which, in conjunction with the size and scale of their construction points to a complex ritual practice. The inclusion of both wild and domestic taxa as votive offerings and the deliberate and specific ritual selection of upper cranial elements, particularly horns, have comparative precedent in other Neolithic ritual behaviours across the Near East [86–92]. Whilst the predominance of male animals (based on the identifiable corpus) and the slaughtering hypothesis may suggest an emphasis on fertility, pasturage and perhaps rain (or water), with the latter also suggested by the placement of the mustatils, often in close association with bodies of water. Furthermore, the positioning of these faunal elements around what appears to be betyl, represents one of the earliest chronometrically dated examples of a tradition which was to become an important feature of the pre-Islamic ritual landscape of Arabia.

Finally, the identification of multiple phases of offerings, albeit over a relatively short period of time, as well as the later inhumation of an individual, who was interred close to the head of the mustatil, may suggest an early form of ‘pilgrimage’ or at the very least shrine revisiting was associated with the mustatil tradition. Like betyls, pilgrimage was to become a hallmark of the religious landscape of the Arabian Peninsula, and based on the aforementioned data it is possible that this phenomenon has its origins earlier than previously supposed, in the Neolithic of north-west Arabia. In summary, the evidence from site IDIHA-0008222 suggests that the mustatil tradition was characterised by the intersection of belief and economic life-ways. The incorporation of these two facets suggests a deeply rooted ideological entanglement, one which was shared over a vast geographic distance, indicating a far more interconnected landscape and culture than had previously been supposed for the Neolithic period in north-west Arabia.
